# Pathogenic role of super-enhancers as potential therapeutic targets in lung cancer

**DOI:** 10.3389/fphar.2024.1383580

**Published:** 2024-04-12

**Authors:** Zhiyuan Yao, Peng Song, Wenjie Jiao

**Affiliations:** ^1^ Department of Thoracic Surgery, The Affiliated Hospital of Qingdao University, Qingdao, China; ^2^ Department of Thoracic Surgery, Beijing Friendship Hospital, Capital Medical University, Beijing, China

**Keywords:** epigenetic modifications, lung cancer, subclassification, super-enhancers, therapeutic targets

## Abstract

Lung cancer is still one of the deadliest malignancies today, and most patients with advanced lung cancer pass away from disease progression that is uncontrollable by medications. Super-enhancers (SEs) are large clusters of enhancers in the genome’s non-coding sequences that actively trigger transcription. Although SEs have just been identified over the past 10 years, their intricate structure and crucial role in determining cell identity and promoting tumorigenesis and progression are increasingly coming to light. Here, we review the structural composition of SEs, the auto-regulatory circuits, the control mechanisms of downstream genes and pathways, and the characterization of subgroups classified according to SEs in lung cancer. Additionally, we discuss the therapeutic targets, several small-molecule inhibitors, and available treatment options for SEs in lung cancer. Combination therapies have demonstrated considerable advantages in preclinical models, and we anticipate that these drugs will soon enter clinical studies and benefit patients.

## 1 Introduction

Lung cancer remains one of the most prevalent and severe tumors, although the mortality risk continues to decline thanks to improvements in early identification and treatment strategies (Siegel et al., 2022). Of all lung cancer histologic subtypes, the most common (85%) is non-small cell lung cancer (NSCLC), with the remaining 10%–15% being small cell lung cancer (SCLC) and other rare cancers (Siegel et al., 2020). Frustratingly, with a 5-year survival rate of just seven percent, SCLC is the most aggressive subtype of lung cancer (Frese et al., 2021). With the advance of targeted medicines and immunotherapies, the long-term prognosis of lung cancer has dramatically improved; however, it is still less than satisfactory for most advanced lung cancers (Wang et al., 2021).

Genetic mutations have been extensively studied in lung cancer, which change coding sequences (e.g., sequences directly coding for oncoproteins) and non-coding sequences (e.g., promoters and enhancers), directly or indirectly favoring clonal proliferation in cancer cells (Lengauer et al., 1998). Current targeted therapies for lung cancer are also mainly based on genetic mutation. However, disturbances in epigenetic regulation can also lead to the occurrence and metastasis of lung cancer (Dong et al., 2023; Kostyrko et al., 2023). For example, the MYC proto-oncogenes family can broadly affect cell proliferation and apoptosis through complex regulatory networks (Carroll et al., 2018). In addition to the copy number increases from genetic mutations, MYC overexpression is also inextricably linked to surrounding regulatory sequences, with a growing number of studies demonstrating that super-enhancers (SEs) act as culprits in this process. Studies have revealed that the mechanism of SE formation involves mutations, and the uniqueness of epigenetic modifications at SEs loci is related to the progression of acute myeloid leukemia (Cao et al., 2022), colorectal cancer (Liu Q. et al., 2022), and esophageal carcinoma (Shi et al., 2022).

Hence, we introduce the structure and function of SEs and summarize the oncogenic mechanism, classifying functions, and potential therapeutic targets and drugs of SEs in lung cancer here.

## 2 Subsections

### 2.1 Identification and structural characterization of SEs

**FIGURE 1:**
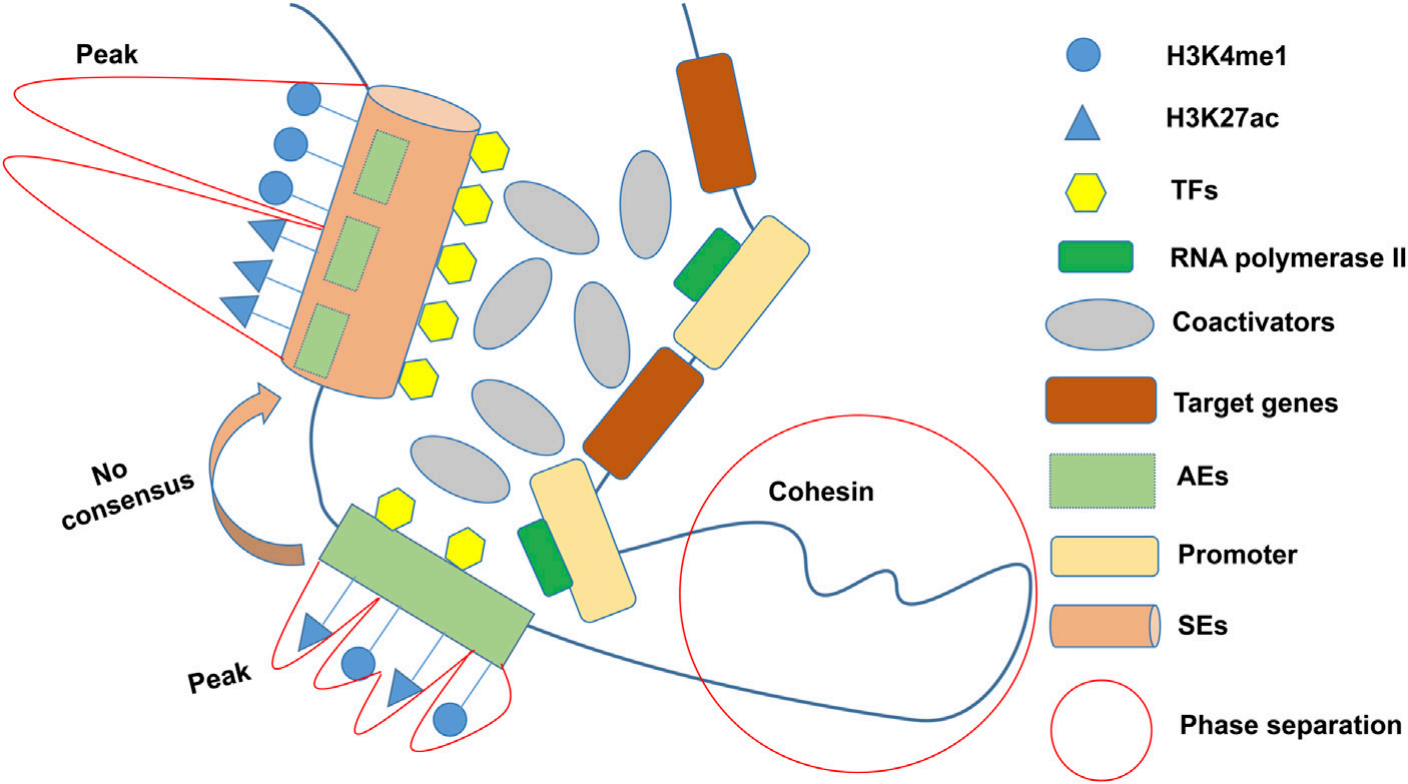
General definition of SEs. SEs characterized by multiple overlapping enrichments (peaks) of H3K4me1 and H3K27ac are usually composed of several AEs, but there is no consensus on the cutoff of peaks. Multiple coactivators cluster around SEs in a phase-separated manner to activate pro-transcriptional functions. TFs: Transcription factors; AEs: Active enhancers; SEs: Super-enhancers.

In the 1980s, a 72 bp sequence, later called “enhancer,” was identified in the genome of the SV40/hemoglobin β1 recombinant gene, accompanied by the overexpression of the rabbit β globin gene (Banerji et al., 1981). Enhancers are cis-regulatory elements that interact with promoters from different positions and distances to increase the expression of controlled genes (Blackwood and Kadonaga, 1998). Richard A. Young identified larger clusters of enhancers, known as SEs (or stretch enhancers), which drove high transcription levels of key genes and defined cell identity in pluripotent embryonic stem cells (ESCs) (Whyte et al., 2013). Researchers have created some online repositories such as dbSUPER (Khan and Zhang, 2016) (https://asntech.org/dbsuper/), SEdb (Jiang et al., 2019; Wang Y. et al., 2023) (https://bio.liclab.net/sedb/), SEanalysis (Qian et al., 2019) (https://bio.liclab.net/SEanalysis/), and SEA (Wei et al., 2016; Chen et al., 2020) (http://sea.edbc.org/) to share SEs (and related genes) and their genetic and epigenetic annotations. The constituent enhancers of SEs are with multiple overlapping enrichments of H3K4me1 and H3K27ac of active enhancers (AEs) marks (Yoo et al., 2019; Barral and Déjardin, 2023) (Figure 1). The Rank Ordering of Super Enhancers (ROSE) algorithm is the most commonly used method to identify SEs (Lovén et al., 2013). The algorithm ranks H3K27ac chromatin immunoprecipitation sequencing (ChIP-Seq) signals and defines enhancers above the inflection point (slope >1) as SEs. However, there is still no consensus on an arbitrary cutoff of enrichment level within certain proximity for considering whether an active enhancer is a super-enhancer. As ChIP-seq has advanced, more and more SEs have been discovered to be involved in carcinogenesis, tumor immune evasion (Xu et al., 2019), and various autoimmune disorders (Parker et al., 2013; Vahedi et al., 2015; Yamagata et al., 2022). It is worth mentioning that other techniques, including 4C-seq (Jiang et al., 2020), Hi-C (Huang J. et al., 2018), ChIA-PET (Dowen et al., 2014), ChIP-STARR-seq (Barakat et al., 2018), GRO-seq (Hah et al., 2015), DNase-seq (Kang et al., 2021), and ATAC-seq (Adam et al., 2020), have also been employed to detect SEs in recent years. Lately, Jialiang Huang’s team refined the hierarchical structure of SEs into hub enhancers and non-hub enhancers by hierarchical scoring (Huang J. et al., 2018). Hub enhancers outperform non-hub enhancers in the interaction with regulatory factors and effects on activating target gene transcription.

As a member of the bromodomain and extra-terminal domain (BET) family and closely associated with SE-related oncogenic transcription, bromodomain-containing protein 4 (BRD4) binds to acetylated modified histones and recruits additional proteins (such as TEFb and Mediator) to indirectly participate in the initiation of transcription and the control of transcriptional elongation (Yang et al., 2005). BET inhibitors (BETi) separate BET proteins from chromatin by competitively binding with histone-acetylated lysines of the BET bromodomain. SEs are highly sensitive to BETi (Fontanals-Cirera et al., 2017). JQ1 is the first reported BETi (Filippakopoulos et al., 2010) and has been widely used for SE identification (Das et al., 2023; Zheng et al., 2024). JQ1 disrupts the transcriptional activity of the master regulators ETS2, HNF4A, and JUNB, suppresses the TGF-induced EMT status, and decreases tumor cell migration and invasion in NSCLC cells (Chang et al., 2016). JQ1, in combination with NSD2 knockdown, inhibits the activation of RAS-driven transcriptional programs in lung cancer cells (García-Carpizo et al., 2016). JQ1 downregulates the expression of HK2, whose promoter is specifically SEs hijacked in a dose-dependent manner in LUAD (Song et al., 2022). In addition, JQ1 exhibits this dose-dependent pharmacological profile in acute myeloid leukemia (Jang et al., 2017), uterine carcinoma (Bonazzoli et al., 2018), and diffuse large B-cell lymphoma (Trabucco et al., 2015).

### 2.2 Transcription factors (TFs) and core TF involved in coding SE-associated genes

Chromatin accessibility allows physical contact between cis-acting elements and trans-acting factors to regulate transcription processes (Klemm et al., 2019). Breast cancer cells undergoing SE reprogramming produced the accessible chromatin structure and had a higher propensity for lung metastasis (Li K. et al., 2019). TFs are proteins that bind to specific DNA sequences (Spitz and Furlong, 2012). If abnormally enriched at oncogene promoters or enhancers, however, they can result in gene overexpression and carcinogenesis (You et al., 2018). SE-driven genes encode core TFs, which can bind to their SEs or others and interact with other core TFs to regulate the expression of additional genes and form cell- or tissue-specific core regulatory circuits (CRC) (Saint-André et al., 2016; Feng C. et al., 2023). For example, the three specifically SE-activated core TFs ELF3, EHF, and TGIF1 constituted the major component of a CRC with an interdependent pattern that tightly regulated cell migration and invasion in lung adenocarcinoma (LUAD) (Zhang T. et al., 2020).

### 2.3 Essential SE-associated transcriptional coactivators

Transcriptional coactivators are a variety of proteins with transcription-related functions. As mentioned above, BRD4 is a crucial part of SEs and plays an essential role in the transcriptional activation of oncogenes. Besides, MED1, Mediator, and CBP/p300 with acetyltransferase activity bind to TFs to assist in the assembly of the transcription preinitiation complex and stabilize the transcriptional activity of RNA polymerase II (Ogryzko et al., 1996; Mao et al., 2019; Rengachari et al., 2021). Benjamin R Sabari’s team proposed that transcriptional coactivators enhance transcriptional activity by forming droplets around SEs through a phase separation process (Sabari et al., 2018). However, whether SE-dependent transcriptional enhancement depends on this mechanism is still debatable (Trojanowski et al., 2022).

Overall, the effects of SEs on transcriptional activation are more forceful than that of typical enhancers (TEs) due to their higher region spanning, higher histone modifications, TFs, and RNA polymerase II enrichment, and more binding sites for transcriptional coactivators (Hnisz et al., 2013; Whyte et al., 2013).

### 2.4 Epigenetic modification associated with SEs

Two key epigenetic regulatory mechanisms involved in SEs are histone modifications (mostly methylation and acetylation) and DNA methylation. Modified histones in the presence of catalytic enzymes (histone lysine methyltransferases or histone acetyltransferases, etc.) can alter chromatin accessibility, affect the assembly of transcriptional complexes, and enhance or reduce the interaction of TFs to gene structures, resulting in altered levels of transcription and translation of downstream genes, which can trigger the development of cancer, autoimmune diseases, and even chronic diseases (Arrowsmith et al., 2012; Fatma et al., 2022; Yang J. et al., 2023). For instance, in a mouse model of LUAD, the deletion of KMT2D (a histone methyltransferase) caused decreased global levels of H3K4me1 and H3K27ac of SEs, leading to the suppression of SE-associated antioncogene Per2 (an antioncogene that inhibits glycolysis) and the activation of glycolytic processes, promoting the growth of cancer (Alam et al., 2020). Another study found that overexpression of NSD2, a histone methyltransferase, resulted in H3K36me2 spreading to the intergenic region of the low H3K27me3 region to exert pro-transcriptional effects (García-Carpizo et al., 2016). That is an example of long-range epigenetic remodeling. It is generally accepted that hypermethylation of CpG islands in the promoter region usually represses gene expression, whereas hypermethylation of gene bodies usually shows the opposite effects (Yang et al., 2014). Interestingly, a study found that highly expressed genes covered by SEs had lower levels of gene body methylation, which resulted in a significant negative correlation between gene body methylation and expression, contrary to popular belief (Pongor et al., 2022). These findings imply an intrinsic link between DNA modification, histone modification, and SEs that jointly influence gene expression.

### 2.5 SEs formation (or translocation) caused by genetic variation in lung cancer

Several studies have elucidated that the formation (or translocation) of SEs is dependent on genetic variants such as single nucleotide variants (SNVs), insertion deletions (indels), and chromosomal structural variants (SVs) (Mansour et al., 2014; Xing et al., 2019; Wang J. et al., 2023). However, the current research discovered only gene fusion or rearrangement was responsible for SEs’ formation in different tissue subtypes of lung cancer. A study found that focal amplified SEs in LUAD acted on the MYC promoter to spark off transcriptional overactivation (Zhang et al., 2016). And it subsequently demonstrated that the mutations arose from a tandem duplication of a non-coding sequence. In LUAD patients, ALK or ROS1 rearrangements triggered SEs hijacking upstream of EML4 and SLC34A2, driving expression of the new oncogenic fusion genes ALK-EML4 and ROS1-SLC34A2, respectively (Yuan et al., 2021). NUT Carcinoma of the Lung is a rare lung cancer subtype of squamous origin that is extremely aggressive and highly associated with NUT gene fusions (Travis et al., 2015). The fusion gene BRD4/3-NUT co-localized with p300, and the corresponding hyperacetylated region showed a similar SE hijacking function to increase the pro-transcriptional effects of BRD4 (Eagen and French, 2021). Adenoid cystic carcinoma (ACC) originates from the secretory glands and is usually molecularly labeled by the MYB-NFIB fusion oncogene (Persson et al., 2009). MYB rearrangements are widely observed in primary pulmonary adenoid cystic carcinoma (PACC) (Roden et al., 2015). A self-regulatory feedback loop was generated when gene rearrangements (e.g., MYB -NFIB, MYB-TGFBR3, MYB-RAD51B gene fusion) occurring in ACC caused SE translocations and overexpression of MYB proteins (Drier et al., 2016).

### 2.6 Carcinogenic roles and regulatory mechanisms of SEs in lung cancers

**FIGURE 2:**
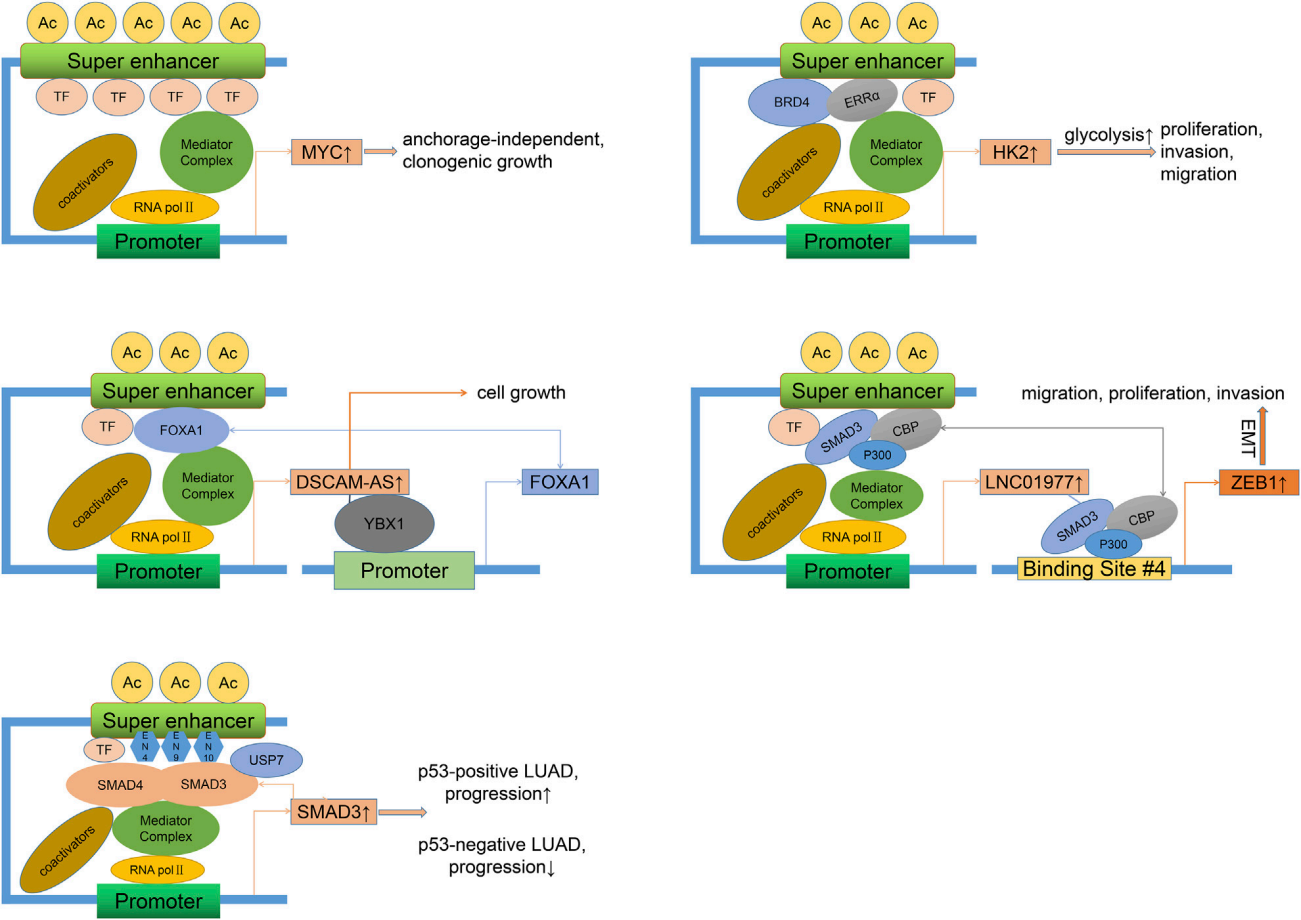
Oncogenic mechanisms of SEs in LUAD. Different regulatory factors can directly (binding to SE motifs) or indirectly (recruiting more activators) lead to the overexpression of SE-related genes and the progression (proliferation, invasion, migration) of LUAD. This process may involve the activation of self-regulatory circuits. LUAD, Lung adenocarcinoma.

Previous studies on SE-associated activation of oncogenes and signaling pathways have mainly focused on LUAD, as shown in Figure 2. A study showed that focal amplification of the SEs 3′to MYC caused increased expression of MYC and other MYC-target genes (Zhang et al., 2016). The master transcription factor ASCL1 regulates the expression of multiple SE-associated oncogenes, such as RET and MYCL (Miyashita et al., 2018). Nuclear transcription factor estrogen-related receptor alpha (ERRα) coordinates with BRD4 to drive the expression of SE-associated glycolytic gene HK2, leading to enhanced glycolysis progress and growth of malignant cells (Song et al., 2022). Specific SE-driven PADI gene families associated with tumor proliferation, invasion, and colony formation were identified as highly expressed in Osimertinib-resistant LUAD cells (Li H. et al., 2022). SE-associated CENPO was highly expressed in LUAD and positively correlated with the tumor stage, which could promote carcinogenesis and progression by regulating the cell cycle and tumor immune microenvironment (Shi et al., 2023).

As we all know, long noncoding RNAs (LncRNAs) are a class of RNAs longer than 200 nt without encoding proteins. Super-enhancer lncRNAs (SE-lncRNAs) are transcribed from super-enhancer genomic regions, which activate the neighboring genes or trans-activate the distant genes through several mechanisms such as transcription factor trapping, promotion of chromatin looping, histone modification, recruitment of phosphorylating RNA polymerase II (Pol II), and isolation of the transcriptional repressor (Schaukowitch et al., 2014; Alvarez-Dominguez et al., 2017; Soibam, 2017; Lee et al., 2020). FOXA1, a driver of multiple cancers (Martínez-Jiménez et al., 2020), can maintain malignant cell survival and promote progression by sensitizing the TGF-β pathway (Wei et al., 2023), activating glycolysis (Xie et al., 2022), and inhibiting autophagy (Li J. et al., 2022). FOXA1 was reported to stimulate LncRNA DSCAM-AS1 at its SEs region (Zhang Y. et al., 2020). DSCAM-AS1, in turn, positively activates FOXA1 expression by interacting with Y box binding protein 1 (YBX1, a DNA/RNA binding protein). Silencing of DSCAM-AS1 inhibits FOXA1 expression and cell growth. Epithelial-mesenchymal transition (EMT), a process by which epithelial cells acquire a mesenchymal cell phenotype and the ability to migrate and invade, is regulated by a series of TFs (Akhmetkaliyev et al., 2023). As a mediator between CDK1 and PRDX1, SE-associated lncRNA LINC00880, activated by TFs FOXP3, leads to phosphorylation of PRDX1 and dysregulation of the PTEN/AKT pathway and metastasis of LUAD (Feng Y. et al., 2023). However, as the LUAD stage progressed, the expression of LINC00880 tended to decrease, and over-expression of LINC00880 had a significant negative prognostic value only in patients with pathological stage I. SEs and related TFs such as ETS2, HNF4A, and JUNB control the high expression of some key EMT genes and define the intermediate state of the EMT process in LUAD cells (Chang et al., 2016). Besides, EMT can be mediated by classical TGF-β/SMAD pathway (Zhang et al., 2014). As one of the receptor-regulated SMAD family members, SMAD3 readily occupies the SE site of LINC01977 and drives its expression in a high TGF-β background (Zhang et al., 2022). In response, LINC01977 boosts the interaction between SMAD3 and CBP/P300 to form a SMAD3/CBP/P300 complex targeting and activating the ZEB1 gene, which induces EMT and causes migration, proliferation, and invasion of LUAD, especially in the early stage of LUAD due to high infiltration of TAM2 (inducing a rich TGF-β environment) and elevated expression of SE-related LINC01977 in this stage. Notably, in p53-deficient lung cancers, the presence of the USP7 (a deubiquitinase) maintains the adherence of the SMAD3 to its own SE elements (EN4, EN9, and EN10), thereby maintaining a positive autoregulatory loop (Huang et al., 2021). However, due to the absence of the p53/MDM2 pathway, USP7 acts as a cancer suppressor. A study showed that TP63-mediated SE-driven LINC01503 was overexpressed and oncogenic in esophageal squamous carcinoma (Xie et al., 2018). Overexpression of LINC01503 was also observed in NSCLC cells and associated with proliferation, migration, and invasion, although it is unclear whether this process is SE-dependent (Shen et al., 2020). Another analysis found that SE-associated lncRNA AC074117.1 was significantly expressed and served as an independent prognostic marker in LUAD (Li et al., 2021).

### 2.7 Differential landscapes of SEs between LUAD and normal tissues

Several studies comparing LUAD cell lines (or cancerous tissues from patients) with normal tissues have revealed marked differences in SEs landscapes. Enrichment analysis of the respective SE-target genes may help to explain normal physiological processes and tumor carcinogenesis and progression to some extent. LUAD-specific SE-regulated genes are involved in the “lung cancer” disease class and series of oncogenic pathways and are strongly associated with transcriptional disorder. In contrast, normal tissue-specific SE-regulated genes are prominent in fundamental cellular structures and activities (Zhang T. et al., 2020; Zhou et al., 2020; Yuan et al., 2021). Interestingly, from one study, only GO terms (like cell-cell adhesion) with significantly lower SEs counts were observed in cancer cells, which suggested that downregulation of “normal” genes that negatively regulated carcinogenesis could also lead to cancers even when cancer-specific SEs “did not work” (Li X. et al., 2019). A bioinformatics study revealed that cancer-specific H3K27Ac peak had a higher percentage of overlap with accessible chromatin regions to favor transcription than H3K27Ac peak of normal-specific (Jiang et al., 2022).

In conclusion, cancer-specific SEs are more susceptible to other regulators and play a crucial role in determining cancer progression.

### 2.8 Tumor heterogeneity in SEs helping in typing of lung cancer

**FIGURE 3:**
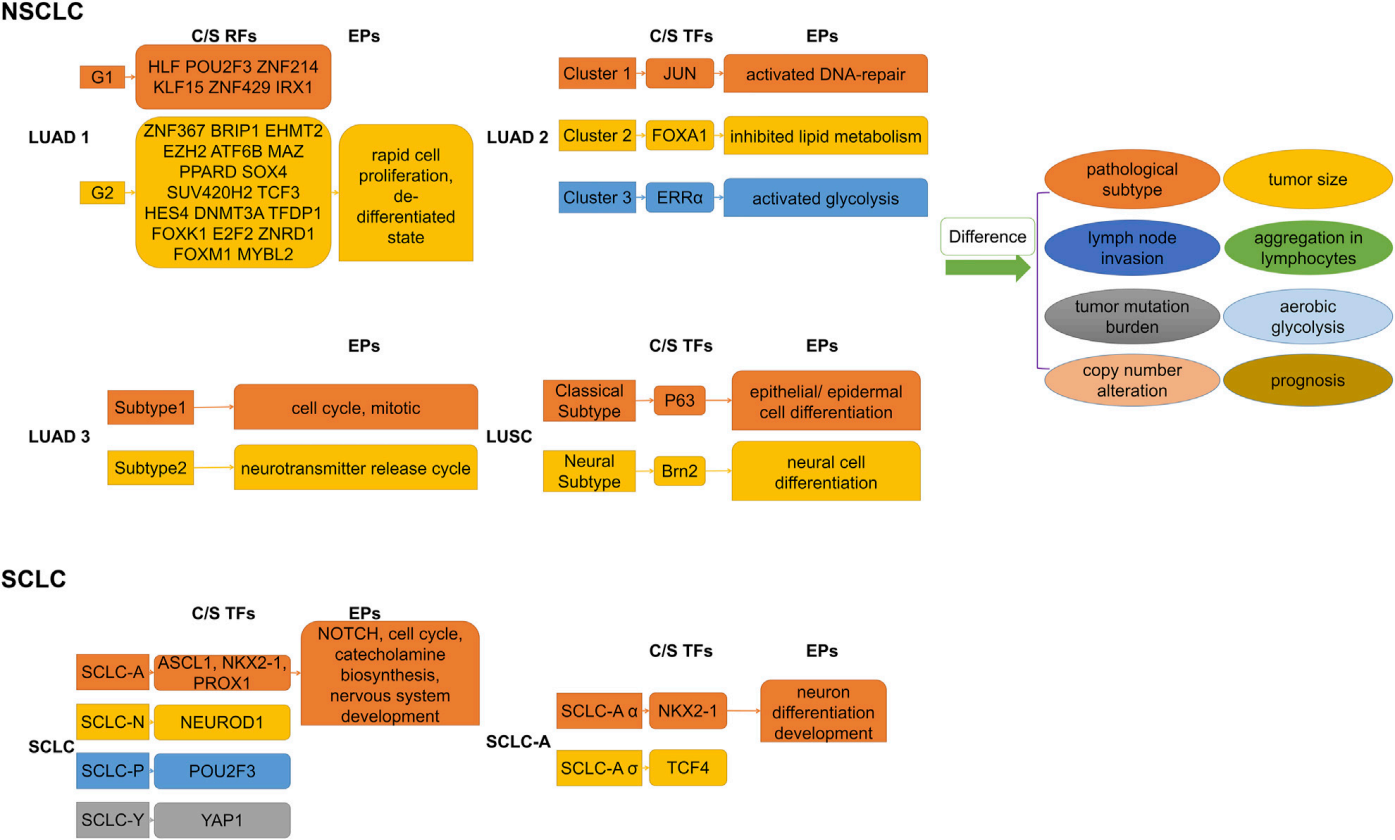
Subdividing lung cancer subtypes by SE-associated regulatory factors. Differences in SE-associated regulatory mechanisms give rise to differential enrichment of SE-regulated gene sets, which explains the differences in biological behaviors of respective subpopulations to some extent. C/S, Core/Specific; RFs, Regulatory factors; TFs, Transcription factors; EPs, Enrichment pathways; NSCLC, Non-small cell lung cancer; SCLC, Small cell lung cancer; LUAD, Lung adenocarcinoma. LUSC, Lung squamous cell carcinoma.

Dentro’s whole genome sequence analysis of 2,658 cancer samples from 38 cancer types from the PCAWG project found that genomic instability allowed tumors to evolve into distinct subclonal populations with different biological behaviors among them (Dentro et al., 2021). Epigenomic diversity and instability are widely observed in heterogeneous subclonal populations of multiple types of cancer, involving differences in chromatin accessibility, methylation levels of gene structure (such as gene bodies, promoters, and enhancers of non-coding sequences), and histone profiles (Ding et al., 2019; Pastore et al., 2019; Cejas et al., 2021; El et al., 2023). The analysis of the H3K27Ac peak can directly clarify the distribution characteristics of SEs and assist in identifying associated tumor heterogeneity with them. Figure 3 illustrates the major regulators of SEs and enrichment pathways of SE-related genes in different subgroups of lung cancer.

### 2.9 High inter-tumor epigenetic heterogeneity presenting in NSCLC

Based on histone modification profiles, a study divided LUAD tissues into two groups that differed significantly in clinical features (e.g., histopathologic subtypes, lymph node invasion), gene mutation patterns, and prognosis (Yuan et al., 2021). Each had its own set of specific core regulatory factors and networks. Specific SE target genes in the poor prognosis group were enriched in invasion- and metastasis-related pathways (e.g., FOXM1, E2F pathway) and dedifferentiated state. In addition, core regulatory networks might shift at early stages, leading to the transition to a terminal stage. Super-enhancer RNA (SeRNA) is known to reflect SE properties and cell specificity (Chen and Liang, 2020; Tu et al., 2021). The distinct groups based on SeRNA profiles showed significant differences in immune infiltration, glycolysis levels, and prognosis (Song et al., 2022). Specifically, tissues in the worst prognostic group had the most chromosomally unstable regions, highest glucose uptake progression, hypoxia-immunotherapy response scores, the most high-risk solid subtype component, and lymph node metastases tendency. A bioinformatics analysis distinguished two subgroups of LUAD with significantly different somatic mutations, survival outcomes, and sensitivities to chemotherapeutic agents by SEs clustering analysis (Jiang et al., 2022). The transcription factor SOX2 usually cooperates with p63 to determine classical subtypes of squamous lung cancer (Watanabe et al., 2014). However, a highly expressed lineage-specific transcription factor Brn2 (an oncogenic factor associated with neuroendocrine carcinomas) was found to replace p63 in lung squamous cell carcinoma (LUSC) cells and to synergize with SOX2 in defining a new “neural” subtype with higher aggressiveness related to poorer prognosis (Sato et al., 2019).

### 2.10 Fine staging of SCLC using SEs as a hot research topic presently

Investigators have found that differential expression of the TFs ASCL1, NEUROD1, POU2F3, and YAP1 defined four molecular subtypes of SCLC, respectively, and that these TFs co-localized with SEs in the corresponding SCLC subtypes (Borromeo et al., 2016; Huang YH. et al., 2018; Rudin et al., 2019). Multiple lineage-specific TFs are also present in different subtypes of SCLC, and subtype switching occurs even in the same tumor (Ireland et al., 2020; Gay et al., 2021). Focusing on the SCLC-A subtype, Karine Pozo found that the TFs ASCL1, NKX2-1, and PROX1 were associated with SEs and that their interactions formed a regulatory network to control Notch signaling (Pozo et al., 2021). On this basis, Ranran Kong subdivided SCLC-A isoforms and discovered that collaborative NKX2-1/SOX1 regulation defined a new, unique SCLC-Aα subtype (Kong et al., 2022). A recent study found that the complex network of ASCL1-regulated SE-associated miRNAs influenced the identification of SCLC molecular subtypes (Miyakawa et al., 2022). SCLC subtypes identified by TFs differ in EMT status, immune gene expression, immunotherapy (or chemotherapy) benefit, and drug resistance (Gay et al., 2021; Qu et al., 2022). Therefore, subdividing the subtypes of SCLC step by step is an important research direction, which helps us better understand its pathogenesis and develop therapeutic approaches.

### 2.11 Potential therapeutic targets and clinical/preclinical trials associated with SEs in lung cancer

In recent years, large-scale, individualized molecular profiling of tumors has led to the discovery of an increasing number of targets and therapeutic options to improve the clinical prognosis of patients (Corces et al., 2018; Wu et al., 2022). Due to an inadequate understanding of etiology and pathogenesis and a lack of identified therapeutic targets, more biomarker-driven trials are required to advance therapeutic technologies (William and Glisson, 2011). Transcription of SE-driven genes is highly sensitive to BRD4 inhibitors since studies reveal BETi preferentially repress transcription of SE-related genes relative to TE-related genes (Lovén et al., 2013). The TFIIH complex subunit cyclin-dependent kinase 7 (CDK7), which controls the cell cycle and initiates transcription by Pol II, has emerged as a brand-new target for treating cancer (Larochelle et al., 2012; Suski et al., 2021). Plus, the catalytic member of the P-TEFb complex, cyclin-dependent kinase 9 (CDK9), promotes the transcriptional elongation of Pol II (Zhou et al., 2012). CDK7 can also phosphorylate to activate CDK9 (Larochelle et al., 2012). Therefore, BET, CDK7, and CDK9 may represent viable therapeutic targets for focusing on SE-dependent transcription. Table 1 demonstrates the clinical trials currently underway or have concluded. Besides, a series of preclinical studies of different drug combinations have laid the groundwork for more novel combination therapeutic regimens to enter clinical trials, as shown in Table 2. We provide a brief overview of the results reported.

**TABLE 1:** SE-related potential drugs in clinical trials for lung cancer.

Target	Compound	Phase	Status	Cancer type	NCT number
BET/BRD4	Birabresib	1	Terminated	NSCLC, NMC	NCT02698176
		1	Completed (Lewin et al., 2018)	NSCLC, NMC	NCT02259114
	PLX-2853	1	Completed	SCLC	NCT03297424
	Mivebresib	1	Completed	NSCLC, SCLC	NCT02391480
	Molibresib	2	Withdrawn	SCLC	NCT03266159
		1	Completed (Cousin et al., 2022)	SCLC, NMC	NCT01587703
	ZEN-3694	2	Recruiting	LUSC	NCT05607108
	INCB-057643	1/2	Terminated	NSCLC	NCT02959437
CDK7	Seliciclib	2	Terminated	NSCLC	NCT00372073
	SY-5609	1	Active, not recruiting	SCLC	NCT04247126
	Roniciclib	2	Terminated (Reck et al., 2019)	SCLC	NCT02161419
		1/2	Withdrawn	NSCLC	NCT02522910
		1/2	Terminated (Cho et al., 2018)	SCLC	NCT01573338
CDK9	Dinaciclib	2	Completed (Stephenson et al., 2014)	NSCLC	NCT00732810
	Hydrochloride	1	Terminated	NSCLC, SCLC	NCT00094978
	PRT-2527	1	Active, not recruiting	NSCLC	NCT05159518

BET, Bromodomain and extra-terminal domain; BRD4, Bromodomain-containing protein 4; CDK, Cyclin-dependent kinase; NSCLC, Non-small cell lung cancer; SCLC, small cell lung cancer; LUSC, lung squamous cell carcinoma; NMC, Nuclear protein in testis (NUT) midline carcinoma.

**TABLE 2:** Combination therapeutic regimens related to SEs in pre-clinical trials for lung cancer.

Agents	Combination	Mechanisms	Cancer type	Malignant features in *vivo* and *in vitro* experiences	References
QCA570	Osimertinib	reduction of Mcl-1	NSCLC	decreased survival, apoptosis, inhibition of formation and colonies growth	Liu et al. (2022b)
ABBV-075	Venetoclax	disruption of Bim-Bcl2 complexes, release of Bim	SCLC	apoptosis, tumor regression	Lam et al. (2017)
JQ1	ABT-263	disruption of Bim-Bcl2 complexes, release of Bim	SCLC	apoptosis	Wang et al. (2017)
GSK525762 (I-BET762)	Talazoparib	inhibition of DNA damage response, HR-DSBR process	SCLC	reduced survival and growth	Fiorentino et al. (2020)
JQ1	Paclitaxel	downregulation of BET protein levels	NSCLC	apoptosis, inhibition of protective autophagy	Zhou et al. (2021)
JQ1, AZD5153, NHWD870	Rapamycin, Everolimus	block of TSC2-mTOR-p70S6K1-BAD pathway	SCLC	intrinsic apoptosis	Kumari et al. (2023)
JQ1	ACY-1215	upregulation of MHC II on tumor cells relies on NK cells	SCLC	apoptosis	Liu et al. (2018)
JQ1, AZD5153	AZD1775	inhibition of NHEJ activity, enhancement of DNA damage, promotion of mitotic entry and mitotic catastrophe	NSCLC	synergistic cytotoxicity, apoptosis, suppression of proliferation	Takashima et al. (2020)

NSCLC, Non-small cell lung cancer; SCLC, small cell lung cancer.

#### 2.11.1 BETi

The BET protein family member BRD4 is currently the focus of much research. We previously discussed the significance of the first-generation BRD4 inhibitor-JQ1 in the discovery and targeted therapy of SEs. In addition to JQ1, more and more small-molecule drugs targeting BET have been prepared and widely employed in clinical trials (Filippakopoulos et al., 2010).

Birabresib (OTX015, MK-8628), a small molecule complex, was first used in hematologic malignancies. In LUAD cells, the master transcriptional regulators ELF3, EHF, and TGIF1 are downregulated in response to JQ1 and OTX015, causing disruption of CRC structure and inhibition of cancer cells’ malignant progression (Zhang T. et al., 2020).

A Phase I study was designed to evaluate the safety and efficacy of single-agent Birabresib in a range of advanced solid tumors (Lewin et al., 2018). Encouragingly, of the nine NSCLC patients with ALK rearrangement or KRAS mutation, seven developed stable disease (SD), and two developed progressive disease (PD). Though thrombocytopenia was one of the serious adverse effects, it was reversible and self-limiting. Another phase 1/2 study assessed the effectiveness and safety of incrementally applied single-agent Molibresib (Cousin et al., 2022). Of the twelve patients with evaluable SCLC, three developed SD, and nine developed PD. However, no clinically meaningful response criteria were observed, as many patients discontinued dose escalation or even reduced the dose due to drug toxicity. The most common treatment‐related adverse events were thrombocytopenia, nausea, and decreased appetite.

The novel BRD4-targeting agent CFT-2718 showed potent anti-tumor activity in xenograft mice. The intensity and duration of apoptosis induction and tumor proliferation inhibition by CFT-2718 surpassed that of the CDK kinase inhibitor dinaciclib in SCLC cells (Sun et al., 2021). OTX015 showed significant anti-cancer activity in NSCLC cells with or without EML4-ALK translocation and EGFR, KRAS mutations (Riveiro et al., 2016). Meanwhile, *in vivo* assays demonstrated that OTX015 significantly inhibited the growth of EML4-ALK (+) NSCLC.

#### 2.11.2 CDK7/9 inhibitors

THZ1 inhibits CDK7 activity in an irreversible covalent binding manner (Kwiatkowski et al., 2014). A study found that both SCLC cell lines and mouse models exhibited high sensitivity to THZ1, as evidenced by considerable tumor proliferation inhibition (Christensen et al., 2014). Genes regulated by master-regulated TFs E2F, NRF1, and CREB are preferentially repressed by THZ1 in SCLC cells, suggesting selective effects of THZ1 on SEs and transcriptional core circuits (Christensen et al., 2014), as in LUAD (Zhang T. et al., 2020), neuroblastoma (Chipumuro et al., 2014), and triple-negative breast cancer (Wang et al., 2015). On the other hand, the low sensitivity and specificity of pan-CDK inhibitors for SCLC demonstrate the significance of covalent binding mechanisms in therapeutic efficacy (Christensen et al., 2014). Since the complex formed by BRD4 binding to CDK9/P-TEFb is localized in the SEs region and stimulates the transcriptional activation of MYC, synergistic inhibition of these two targets can effectively halt the progression of cancer (Lovén et al., 2013; Lu et al., 2015).

A phase 2 study compared the difference in efficacy between dinaciclib (a CDK9 inhibitor) and erlotinib in previously treated NSCLC patients (Stephenson et al., 2014). Unfortunately, no patients in the dinaciclib group showed objective responses (OR). The main adverse effects of intravenous denazanil were myelosuppression and gastrointestinal toxicity.

YPN-005 is a novel and selective CDK7 inhibitor with super anti-cancer activity compared to conventional THZ1. YPN-005 showed powerful antiproliferative effects in chemotherapy-resistant SCLC cells, patient-derived organoids, and xenograft mice (Choi et al., 2021). No significant toxic reactions were observed in the mice model. 21e is a highly effective and selective CDK9 inhibitor. After receiving 21e, the proportion of NSCLC side population cells (which indicate stem cell potential) drastically decreased (Wang et al., 2019). A reduction of the cancer stemness marker Oct4, apoptosis of cancer cells, and inhibition of proliferation were observed in xenograft mice treated with 21e.

#### 2.11.3 Combination therapy

It is evident that existing clinical trials, although affirming the therapeutic feasibility of BETi and CDK kinase inhibitors, still suffer from ineffectiveness, inefficacy, toxicity, and resistance. Single-agent (or even partial combination) treatment in these trials does not meet the desired standard. Future studies may favor multiple combination strategies to enhance therapeutic efficacy and reduce toxicity.

Roniclib is a pan-CDK inhibitor (including both CDK7 and CDK9). A phase I/II study reported the potential feasibility of Roniclib in combination with cisplatin/carboplatin-etoposide for extensive SCLC (Cho et al., 2018). Chemotherapeutic agents did not alter the pharmacokinetics of Roniclib but reduced Roniclib exposure by more than one-third. Although none of the 46 patients receiving different doses achieved a complete response (CR), 35 achieved a partial response (PR), five achieved SD, and two developed PD. The most common adverse reactions were nausea, vomiting, anemia, and decreased neutrophil count. This study reflects the favorable efficacy and tolerability of Roniclib for SCLC in all. However, another phase II study comparing the efficacy and safety of Roniciclib in combination with platinum-based chemotherapy *versus* chemotherapy alone in patients with extensive SCLC did not give us encouraging results (Reck et al., 2019). Although one patient in the combination therapy group achieved CR, overall, the combination therapy group had a lower objective response rate (ORR) and more treatment-emergent adverse events than the platinum-based chemotherapy group. Common adverse reactions were similar to the previous study (Cho et al., 2018). Nausea was the primary cause of discontinuation in the combined therapy group.

Besides, BET- and CDK-targeted inhibitors demonstrate convincing anti-tumor effects in preclinical models and provide paradigms for clinical trials.

BETi can induce cancer cells toward apoptosis by regulating the balance between pro- and anti-apoptotic proteins. QCA570 is a novel BET degrader that caused the degradation of anti-apoptosis-related proteins Mcl-1 in NSCLC cells (Liu C. et al., 2022). QCA570, in combination with ositinib, induced apoptosis and inhibited the proliferation of ositinib-resistant cells in vivo and *in vitro* experiments. Another study found that the antitumoral activity of the BET inhibitor ABBV-075 in SCLC was partially dependent on the inhibition of BCL2, an anti-apoptotic protein (Lam et al., 2017). *In vivo* and *in vitro* assays confirmed the synergistic antitumor effects of ABBV-075 with the BCL2 inhibitor venetoclax (ABT-199), particularly in tumors with a high level of BCL2 expression. Furthermore, combination treatment with JQ1 and the BCL2 inhibitors ABT-263 damaged the balance of Bim and Bcl-2, resulting in the release of the pro-apoptotic protein BIM in MYCN-amplified SCLC cell lines and exerted growth-inhibitory effects in MYCN-positive xenografts (Wang et al., 2017). Another study similarly found that BET inhibitor I-BET762 combined with the PARP inhibitor talazoparib showed higher antitumoral activity in SCLC cells with elevated MYC expression or amplification of MYCs (Fiorentino et al., 2020). The above research implies that the combined strategy boosts the inhibitory effects of BETi on the MYC-related transcriptional pathway.

BET inhibitor treatment alone may result in compensatory alterations that can lower efficacy or develop drug resistance. Therefore, inhibiting these compensatory changes can improve efficacy. For instance, one investigation discovered that JQ1 alone compensatorily upregulated the BET protein (BRD2,3,4), triggered autophagy, and prevented apoptosis in NSCLC cells (Zhou et al., 2021). JQ1 with the chemotherapeutic agent paclitaxel reversed this upregulation and induced apoptosis. Another study found that BETi upregulate the mTOR upstream kinase RSK3, leading to resistance to apoptosis in SCLC cells (Kumari et al., 2023). The combination of BETi and mTOR inhibitors disrupted mTOR signaling and displayed superior anti-cancer activity than any single drug in vitro (JQ1/rapamycin) and *in vivo* (NHWD870/everolimus) assays.

A combination of inhibitors targeting epigenetic regulators histone deacetylase (HDAC) and BET exerted synergistic antitumoral effects in pancreatic cancer (He et al., 2020), glioblastoma (Gusyatiner et al., 2021), and medulloblastoma (Kling et al., 2022). In SCLC xenograft mice, JQ1 and the histone deacetylase 6 (HDAC6) inhibitor ACY-1215 combined to exert anti-tumor activity dependent on the presence of NK cells (Liu et al., 2018).

Furthermore, JQ1 enhanced DNA double-strand breaks induced by the WEE1 inhibitor AZD1775 and led to mitotic catastrophe, which synergistically produced cytotoxicity and inhibited NSCLC growth in vitro and *in vivo* experiments (Takashima et al., 2020).

In conclusion, in both *in vivo* and *in vitro* studies, whether for SCLC or NSCLC, combination therapies have achieved greater success than any single agent. Combination therapies are also effective in reducing drug resistance and side effects. Based on in-depth studies of more complex carcinogenic pathways, we suppose that more clinical trials will be undertaken in the future.

#### 2.11.4 Other drug candidates in the pan-cancer field

Furthermore, epigenetic modifiers CBP/p300 and HDAC dynamically regulate H3K27 acetylation levels and are potential drug targets in the pan-cancer field (Wang et al., 2022).

CBP/p300 is a histone acetyltransferase that deposits extensive H3K27ac marks and highly occupies SEs (Wang et al., 2022). A study found that the CBP/p300 inhibitor ICG-001 alone upregulated the expression of SE-related oncogenes in glioblastomas, whereas this deleterious effect disappeared when combined with JQ1 (Wiese et al., 2020). Anti-cancer studies with SE-related CBP/p300 inhibitors have been conducted in hematologic malignant neoplasms such as multiple myeloma, acute myeloid leukemia, and non-Hodgkin’s lymphoma (Lasko et al., 2017), as well as in castration-resistant prostate cancer (Chen et al., 2022). Notably, Zhang T et al. found that the EP300 inhibitor CBP30 inhibited the expression of master TFs in LUAD cell lines (Zhang T. et al., 2020).

Studies have confirmed that HDAC inhibitors (HDACi) effectively inhibit the function of SEs (Nagaraja et al., 2017; Sanchez et al., 2018; Nguyen et al., 2020). HDACi targeting SEs exerted great anti-tumor potential in glioblastoma (Nguyen et al., 2020), rhabdomyosarcoma (Gryder et al., 2019), multiple myeloma (Alaterre et al., 2022), stem-like breast cancer (Caslini et al., 2019), ovarian cancer (Shi et al., 2019; Wu et al., 2023), and esophageal squamous cell carcinoma (Jiang et al., 2020). Numerous preclinical studies have confirmed that HDACi inhibits the proliferation of lung cancer cells and is strongly associated with drug resistance (Huang et al., 2014; Wu et al., 2020; He et al., 2022; Eichner et al., 2023). However, it is not yet clear whether this effect is dependent on SEs. Unfortunately, in clinical trials for lung cancer, HDACi are usually used in synergy with radiotherapy, immunotherapy, or epigenetic therapy, as only modest single-agent activity has been demonstrated (Bartling et al., 2005; Traynor et al., 2009; Costa et al., 2014). In any case, drug trials against more SE targets are warranted and expected to provide additional treatment options for lung cancer patients.

## 3 Conclusion

Since their discovery, SEs have received attention in various fields, and the specific functions they exert in different tumor types are gradually being revealed. The basic structure and function of SEs are similar in different malignant tumors, and all are closely related to cancer progression and aggressiveness. However, it is still to be demonstrated that there are many differences in the SEs characteristics of different cancer types: 1. SEs might be widespread in almost all cancer MYC regions (Zhou and Parsons, 2023), whereas studies of SE changes in the MYC region in lung cancer are limited (Zhang et al., 2016). 2. Gene rearrangements/fusions underlie the formation of cancer-specific SEs. However, compared to hematologic tumors, where high-frequency gene fusions are present, rearrangements/fusions that form SEs are less frequently observed in lung cancer (especially squamous lung cancer) (Li et al., 2016). 3. SE-lncRNA might be widespread in pan-cancer (Ropri et al., 2021; Yan et al., 2021; Chuang et al., 2022; Yang Z. et al., 2023; Hu et al., 2023)。SE-lncRNA has been intensively studied in lung cancer (Xie et al., 2018; Shen et al., 2020; Zhang et al., 2022), hepatocellular carcinoma (Peng et al., 2019; Li et al., 2023; Su et al., 2023; Yuan et al., 2023), and gliomas (Bian et al., 2021; Yang Z. et al., 2023; Chen et al., 2023) but has been rarely reported in hematologic tumors (Handa et al., 2020). However, SEs still cannot be accurately identified due to the low specificity of the classical ChIP-seq technique and the technical imperfections of the Rose algorithm. In addition, the molecular mechanism of how oncogenic signaling alters super enhancer-promoter interactions is not yet understood. For example, how do oncogenic signals promote the aggregation of phase-separated condensates near SEs and drive transcription? How do these signals reshape the three-dimensional folding of the genome, altering the otherwise insulating relationships between regulatory elements and pinpointing the timing, location, and level of transcription?

Here, we summarize the tissue specificity of SEs, mechanisms of SEs formation, oncogenic effects, therapeutic targets, and clinical trials in lung cancer.

Researchers identified subgroups of lung tumors with distinctly diverse biological traits (such as aggressiveness) and prognoses using the Rose algorithms and unsupervised clustering, indicating that these groupings have unique oncogenic signaling networks. However, to date, SEs formed by point mutations or indels have not been identified in the lung cancer genome. Moreover, it is difficult to determine the sequential relationship between the activation of oncogenic pathways and the formation of SEs. Unfortunately, only a few studies related to SEs were conducted for squamous lung cancer. It is challengeable and valuable for researchers to accurately identify SE-driven oncogenic mutations of squamous cell carcinoma, given the close connection between smoking and squamous cell carcinoma and the large number of non-oncogenic passenger mutations resulting from long-term exposure to tobacco carcinogens. BETi and CDK kinase inhibitors are the predominant epigenetic agents targeting SEs. However, existing clinical trials of these drugs are less encouraging due to lack of specificity, development of resistance, and the inevitability of toxicity. Nonetheless, many studies in preclinical models have confirmed the potential benefits of combination therapies and suggested that drug combinations with multiple different mechanisms are an area to explore in the future. It is worth mentioning that the body’s immunological status and the tumor microenvironment are unquestionably significant factors influencing medication action, even though the precise mechanism is still under investigation. Epigenetic therapies can remodel the immune microenvironment, and their use in conjunction with immunosuppressive agents is currently a hot topic in cancer care (Chou et al., 2020; Luo et al., 2022; Tien et al., 2023). In addition to drug therapy, gene editing techniques (e.g., CRISPR/Cas9) can specifically knock down SEs of interest to prevent activation and cascade amplification of oncogenic transcripts. For instance, the knockdown of e3, one of the key components of SEs in the neighboring non-coding region of MYC, downregulates the expression of MYC and downstream targets and inhibits the clonal growth of LUAD cells (Zhang et al., 2016). Therefore, pinpointing functional components within SEs clusters may provide insight into precision medicine. In conclusion, further research into the oncogenic mechanism of SEs will aid in the development of personalized therapeutic strategies and the long-term survival of lung cancer patients.

## References

[B1] Adam R. C. Yang H. Ge Y. Infarinato N. R. Gur-Cohen S. Miao Y. (2020). NFI transcription factors provide chromatin access to maintain stem cell identity while preventing unintended lineage fate choices. Nat. Cell Biol. 22 (6), 640–650. 10.1038/s41556-020-0513-0 32393888 PMC7367149

[B2] Akhmetkaliyev A. Alibrahim N. Shafiee D. Tulchinsky E. (2023). EMT/MET plasticity in cancer and Go-or-Grow decisions in quiescence: the two sides of the same coin? Mol. Cancer 22 (1), 90. 10.1186/s12943-023-01793-z 37259089 PMC10230810

[B3] Alam H. Tang M. Maitituoheti M. Dhar S. S. Kumar M. Han C. Y. (2020). KMT2D deficiency impairs super-enhancers to confer a glycolytic vulnerability in lung cancer. Cancer Cell 37 (4), 599–617. 10.1016/j.ccell.2020.03.005 32243837 PMC7178078

[B4] Alaterre E. Ovejero S. Herviou L. de Boussac H. Papadopoulos G. Kulis M. (2022). Comprehensive characterization of the epigenetic landscape in Multiple Myeloma. Theranostics 12 (4), 1715–1729. 10.7150/thno.54453 35198065 PMC8825580

[B5] Alvarez-Dominguez J. R. Knoll M. Gromatzky A. A. Lodish H. F. (2017). The super-enhancer-derived alncRNA-EC7/bloodlinc potentiates red blood cell development in trans. Cell Rep. 19 (12), 2503–2514. 10.1016/j.celrep.2017.05.082 28636939 PMC6013260

[B6] Arrowsmith C. H. Bountra C. Fish P. V. Lee K. Schapira M. (2012). Epigenetic protein families: a new frontier for drug discovery. Nat. Rev. Drug Discov. 11 (5), 384–400. 10.1038/nrd3674 22498752

[B7] Banerji J. Rusconi S. Schaffner W. (1981). Expression of a beta-globin gene is enhanced by remote SV40 DNA sequences. Cell 27 (2 Pt 1), 299–308. 10.1016/0092-8674(81)90413-x 6277502

[B8] Barakat T. S. Halbritter F. Zhang M. Rendeiro A. F. Perenthaler E. Bock C. (2018). Functional dissection of the enhancer repertoire in human embryonic stem cells. Cell Stem Cell 23 (2), 276–288. 10.1016/j.stem.2018.06.014 30033119 PMC6084406

[B9] Barral A. Déjardin J. (2023). The chromatin signatures of enhancers and their dynamic regulation. Nucleus 14 (1), 2160551. 10.1080/19491034.2022.2160551 36602897 PMC9828845

[B10] Bartling B. Hofmann H. S. Boettger T. Hansen G. Burdach S. Silber R. E. (2005). Comparative application of antibody and gene array for expression profiling in human squamous cell lung carcinoma. Lung Cancer 49 (2), 145–154. 10.1016/j.lungcan.2005.02.006 16022908

[B11] Bian E. Chen X. Cheng L. Cheng M. Chen Z. Yue X. (2021). Super-enhancer-associated TMEM44-AS1 aggravated glioma progression by forming a positive feedback loop with Myc. J. Exp. Clin. Cancer Res. 40 (1), 337. 10.1186/s13046-021-02129-9 34696771 PMC8543865

[B12] Blackwood E. M. Kadonaga J. T. (1998). Going the distance: a current view of enhancer action. Science 281 (5373), 60–63. 10.1126/science.281.5373.60 9679020

[B13] Bonazzoli E. Predolini F. Cocco E. Bellone S. Altwerger G. Menderes G. (2018). Inhibition of BET bromodomain proteins with GS-5829 and GS-626510 in uterine serous carcinoma, a biologically aggressive variant of endometrial cancer. Clin. Cancer Res. 24 (19), 4845–4853. 10.1158/1078-0432.Ccr-18-0864 29941483 PMC6168417

[B14] Borromeo M. D. Savage T. K. Kollipara R. K. He M. Augustyn A. Osborne J. K. (2016). ASCL1 and NEUROD1 reveal heterogeneity in pulmonary neuroendocrine tumors and regulate distinct genetic programs. Cell Rep. 16 (5), 1259–1272. 10.1016/j.celrep.2016.06.081 27452466 PMC4972690

[B15] Cao Z. Shu Y. Wang J. Wang C. Feng T. Yang L. (2022). Super enhancers: pathogenic roles and potential therapeutic targets for acute myeloid leukemia (AML). Genes. Dis. 9 (6), 1466–1477. 10.1016/j.gendis.2022.01.006 36157504 PMC9485276

[B16] Carroll P. A. Freie B. W. Mathsyaraja H. Eisenman R. N. (2018). The MYC transcription factor network: balancing metabolism, proliferation and oncogenesis. Front. Med. 12 (4), 412–425. 10.1007/s11684-018-0650-z 30054853 PMC7358075

[B17] Caslini C. Hong S. Ban Y. J. Chen X. S. Ince T. A. (2019). HDAC7 regulates histone 3 lysine 27 acetylation and transcriptional activity at super-enhancer-associated genes in breast cancer stem cells. Oncogene 38 (39), 6599–6614. 10.1038/s41388-019-0897-0 31375747

[B18] Cejas P. Xie Y. Font-Tello A. Lim K. Syamala S. Qiu X. (2021). Subtype heterogeneity and epigenetic convergence in neuroendocrine prostate cancer. Nat. Commun. 12 (1), 5775. 10.1038/s41467-021-26042-z 34599169 PMC8486778

[B19] Chang H. Liu Y. Xue M. Liu H. Du S. Zhang L. (2016). Synergistic action of master transcription factors controls epithelial-to-mesenchymal transition. Nucleic Acids Res. 44 (6), 2514–2527. 10.1093/nar/gkw126 26926107 PMC4824118

[B20] Chen C. Zhou D. Gu Y. Wang C. Zhang M. Lin X. (2020). SEA version 3.0: a comprehensive extension and update of the Super-Enhancer archive. Nucleic Acids Res. 48 (D1), D198-D203–d203. 10.1093/nar/gkz1028 31667506 PMC7145603

[B21] Chen H. Liang H. (2020). A high-resolution map of human enhancer RNA loci characterizes super-enhancer activities in cancer. Cancer Cell 38 (5), 701–715. 10.1016/j.ccell.2020.08.020 33007258 PMC7658066

[B22] Chen Q. Yang B. Liu X. Zhang X. D. Zhang L. Liu T. (2022). Histone acetyltransferases CBP/p300 in tumorigenesis and CBP/p300 inhibitors as promising novel anticancer agents. Theranostics 12 (11), 4935–4948. 10.7150/thno.73223 35836809 PMC9274749

[B23] Chen Z. Tian D. Chen X. Cheng M. Xie H. Zhao J. (2023). Super-enhancer-driven lncRNA LIMD1-AS1 activated by CDK7 promotes glioma progression. Cell Death Dis. 14 (6), 383. 10.1038/s41419-023-05892-z 37385987 PMC10310775

[B24] Chipumuro E. Marco E. Christensen C. L. Kwiatkowski N. Zhang T. Hatheway C. M. (2014). CDK7 inhibition suppresses super-enhancer-linked oncogenic transcription in MYCN-driven cancer. Cell 159 (5), 1126–1139. 10.1016/j.cell.2014.10.024 25416950 PMC4243043

[B25] Cho B. C. Dy G. K. Govindan R. Kim D. W. Pennell N. A. Zalcman G. (2018). Phase Ib/II study of the pan-cyclin-dependent kinase inhibitor roniciclib in combination with chemotherapy in patients with extensive-disease small-cell lung cancer. Lung Cancer 123, 14–21. 10.1016/j.lungcan.2018.04.022 30089585

[B26] Choi Y. J. Lee H. Kim D. S. Kim D. H. Kang M. H. Cho Y. H. (2021). Discovery of a novel CDK7 inhibitor YPN-005 in small cell lung cancer. Eur. J. Pharmacol. 907, 174298. 10.1016/j.ejphar.2021.174298 34224696

[B27] Chou J. Quigley D. A. Robinson T. M. Feng F. Y. Ashworth A. (2020). Transcription-associated cyclin-dependent kinases as targets and biomarkers for cancer therapy. Cancer Discov. 10 (3), 351–370. 10.1158/2159-8290.Cd-19-0528 32071145

[B28] Christensen C. L. Kwiatkowski N. Abraham B. J. Carretero J. Al-Shahrour F. Zhang T. (2014). Targeting transcriptional addictions in small cell lung cancer with a covalent CDK7 inhibitor. Cancer Cell 26 (6), 909–922. 10.1016/j.ccell.2014.10.019 25490451 PMC4261156

[B29] Chuang T. D. Quintanilla D. Boos D. Khorram O. (2022). Differential expression of super-enhancer-associated long non-coding RNAs in uterine leiomyomas. Reprod. Sci. 29 (10), 2960–2976. 10.1007/s43032-022-00981-4 35641855 PMC9537225

[B30] Corces M. R. Granja J. M. Shams S. Louie B. H. Seoane J. A. Zhou W. (2018). The chromatin accessibility landscape of primary human cancers. Science 362 (6413), eaav1898. 10.1126/science.aav1898 30361341 PMC6408149

[B31] Costa C. Molina M. A. Drozdowskyj A. Giménez-Capitán A. Bertran-Alamillo J. Karachaliou N. (2014). The impact of EGFR T790M mutations and BIM mRNA expression on outcome in patients with EGFR-mutant NSCLC treated with erlotinib or chemotherapy in the randomized phase III EURTAC trial. Clin. Cancer Res. 20 (7), 2001–2010. 10.1158/1078-0432.Ccr-13-2233 24493829

[B32] Cousin S. Blay J. Y. Garcia I. B. de Bono J. S. Le Tourneau C. Moreno V. (2022). Safety, pharmacokinetic, pharmacodynamic and clinical activity of molibresib for the treatment of nuclear protein in testis carcinoma and other cancers: results of a Phase I/II open-label, dose escalation study. Int. J. Cancer 150 (6), 993–1006. 10.1002/ijc.33861 34724226

[B33] Das N. D. Chang J. C. Hon C. C. Kelly S. T. Ito S. Lizio M. (2023). Defining super-enhancers by highly ranked histone H4 multi-acetylation levels identifies transcription factors associated with glioblastoma stem-like properties. BMC Genomics 24 (1), 574. 10.1186/s12864-023-09659-w 37759202 PMC10523799

[B34] Dentro S. C. Leshchiner I. Haase K. Tarabichi M. Wintersinger J. Deshwar A. G. (2021). Characterizing genetic intra-tumor heterogeneity across 2,658 human cancer genomes. Cell 184 (8), 2239–2254.e39. 10.1016/j.cell.2021.03.009 33831375 PMC8054914

[B35] Ding X. He M. Chan A. W. H. Song Q. X. Sze S. C. Chen H. (2019). Genomic and epigenomic features of primary and recurrent hepatocellular carcinomas. Gastroenterology 157 (6), 1630–1645. 10.1053/j.gastro.2019.09.005 31560893

[B36] Dong Y. Hu H. Zhang X. Zhang Y. Sun X. Wang H. (2023). Phosphorylation of PHF2 by AMPK releases the repressive H3K9me2 and inhibits cancer metastasis. Signal Transduct. Target Ther. 8 (1), 95. 10.1038/s41392-022-01302-6 36872368 PMC9986243

[B37] Dowen J. M. Fan Z. P. Hnisz D. Ren G. Abraham B. J. Zhang L. N. (2014). Control of cell identity genes occurs in insulated neighborhoods in mammalian chromosomes. Cell 159 (2), 374–387. 10.1016/j.cell.2014.09.030 25303531 PMC4197132

[B38] Drier Y. Cotton M. J. Williamson K. E. Gillespie S. M. Ryan R. J. Kluk M. J. (2016). An oncogenic MYB feedback loop drives alternate cell fates in adenoid cystic carcinoma. Nat. Genet. 48 (3), 265–272. 10.1038/ng.3502 26829750 PMC4767593

[B39] Eagen K. P. French C. A. (2021). Supercharging BRD4 with NUT in carcinoma. Oncogene 40 (8), 1396–1408. 10.1038/s41388-020-01625-0 33452461 PMC7914217

[B40] Eichner L. J. Curtis S. D. Brun S. N. McGuire C. K. Gushterova I. Baumgart J. T. (2023). HDAC3 is critical in tumor development and therapeutic resistance in Kras-mutant non-small cell lung cancer. Sci. Adv. 9 (11), eadd3243. 10.1126/sciadv.add3243 36930718 PMC10022903

[B41] El K. L. Y. Pan X. Hlady R. A. Wagner R. T. Shaikh S. Wang L. (2023). Extensive intratumor regional epigenetic heterogeneity in clear cell renal cell carcinoma targets kidney enhancers and is associated with poor outcome. Clin. Epigenetics 15 (1), 71. 10.1186/s13148-023-01471-3 37120552 PMC10149001

[B42] Fatma H. Maurya S. K. Siddique H. R. (2022). Epigenetic modifications of c-MYC: role in cancer cell reprogramming, progression and chemoresistance. Semin. Cancer Biol. 83, 166–176. 10.1016/j.semcancer.2020.11.008 33220458

[B43] Feng C. Song C. Jiang Y. Zhao J. Zhang J. Wang Y. (2023a). Landscape and significance of human super enhancer-driven core transcription regulatory circuitry. Mol. Ther. Nucleic Acids 32, 385–401. 10.1016/j.omtn.2023.03.014 37131406 PMC10149290

[B44] Feng Y. Zhang T. Zhang Z. Liang Y. Wang H. Chen Y. (2023b). The super-enhancer-driven lncRNA LINC00880 acts as a scaffold between CDK1 and PRDX1 to sustain the malignance of lung adenocarcinoma. Cell Death Dis. 14 (8), 551. 10.1038/s41419-023-06047-w 37620336 PMC10449921

[B45] Filippakopoulos P. Qi J. Picaud S. Shen Y. Smith W. B. Fedorov O. (2010). Selective inhibition of BET bromodomains. Nature 468 (7327), 1067–1073. 10.1038/nature09504 20871596 PMC3010259

[B46] Fiorentino F. P. Marchesi I. Schröder C. Schmidt R. Yokota J. Bagella L. (2020). BET-inhibitor I-BET762 and PARP-inhibitor talazoparib synergy in small cell lung cancer cells. Int. J. Mol. Sci. 21 (24), 9595. 10.3390/ijms21249595 33339368 PMC7766292

[B47] Fontanals-Cirera B. Hasson D. Vardabasso C. Di Micco R. Agrawal P. Chowdhury A. (2017). Harnessing BET inhibitor sensitivity reveals AMIGO2 as a melanoma survival gene. Mol. Cell 68 (4), 731–744. 10.1016/j.molcel.2017.11.004 29149598 PMC5993436

[B48] Frese K. K. Simpson K. L. Dive C. (2021). Small cell lung cancer enters the era of precision medicine. Cancer Cell 39 (3), 297–299. 10.1016/j.ccell.2021.02.002 33577787

[B49] García-Carpizo V. Sarmentero J. Han B. Graña O. Ruiz-Llorente S. Pisano D. G. (2016). NSD2 contributes to oncogenic RAS-driven transcription in lung cancer cells through long-range epigenetic activation. Sci. Rep. 6, 32952. 10.1038/srep32952 27604143 PMC5015087

[B50] Gay C. M. Stewart C. A. Park E. M. Diao L. Groves S. M. Heeke S. (2021). Patterns of transcription factor programs and immune pathway activation define four major subtypes of SCLC with distinct therapeutic vulnerabilities. Cancer Cell 39 (3), 346–360.e7. 10.1016/j.ccell.2020.12.014 33482121 PMC8143037

[B51] Gryder B. E. Pomella S. Sayers C. Wu X. S. Song Y. Chiarella A. M. (2019). Histone hyperacetylation disrupts core gene regulatory architecture in rhabdomyosarcoma. Nat. Genet. 51 (12), 1714–1722. 10.1038/s41588-019-0534-4 31784732 PMC6886578

[B52] Gusyatiner O. Bady P. Pham M. D. T. Lei Y. Park J. Daniel R. T. (2021). BET inhibitors repress expression of interferon-stimulated genes and synergize with HDAC inhibitors in glioblastoma. Neuro Oncol. 23 (10), 1680–1692. 10.1093/neuonc/noab115 33987681 PMC8485441

[B53] Hah N. Benner C. Chong L. W. Yu R. T. Downes M. Evans R. M. (2015). Inflammation-sensitive super enhancers form domains of coordinately regulated enhancer RNAs. Proc. Natl. Acad. Sci. U. S. A. 112 (3), E297–E302. 10.1073/pnas.1424028112 25564661 PMC4311831

[B54] Handa H. Honma K. Oda T. Kobayashi N. Kuroda Y. Kimura-Masuda K. (2020). Long noncoding RNA PVT1 is regulated by bromodomain protein BRD4 in multiple myeloma and is associated with disease progression. Int. J. Mol. Sci. 21 (19), 7121. 10.3390/ijms21197121 32992461 PMC7583953

[B55] He S. Dong G. Li Y. Wu S. Wang W. Sheng C. (2020). Potent dual BET/HDAC inhibitors for efficient treatment of pancreatic cancer. Angew. Chem. Int. Ed. Engl. 59 (8), 3028–3032. 10.1002/anie.201915896 31943585

[B56] He T. Gao Y. Fang Y. Zhang Y. Zhang S. Nan F. (2022). The HDAC inhibitor GCJ-490A suppresses c-Met expression through IKKα and overcomes gefitinib resistance in non-small cell lung cancer. Cancer Biol. Med. 19 (8), 1172–1192. 10.20892/j.issn.2095-3941.2021.0130 35188360 PMC9425179

[B57] Hnisz D. Abraham B. J. Lee T. I. Lau A. Saint-André V. Sigova A. A. (2013). Super-enhancers in the control of cell identity and disease. Cell 155 (4), 934–947. 10.1016/j.cell.2013.09.053 24119843 PMC3841062

[B58] Hu X. Wu J. Feng Y. Ma H. Zhang E. Zhang C. (2023). METTL3-stabilized super enhancers-lncRNA SUCLG2-AS1 mediates the formation of a long-range chromatin loop between enhancers and promoters of SOX2 in metastasis and radiosensitivity of nasopharyngeal carcinoma. Clin. Transl. Med. 13 (9), e1361. 10.1002/ctm2.1361 37658588 PMC10474317

[B59] Huang J. Li K. Cai W. Liu X. Zhang Y. Orkin S. H. (2018a). Dissecting super-enhancer hierarchy based on chromatin interactions. Nat. Commun. 9 (1), 943. 10.1038/s41467-018-03279-9 29507293 PMC5838163

[B60] Huang W. J. Tang Y. A. Chen M. Y. Wang Y. J. Hu F. H. Wang T. W. (2014). A histone deacetylase inhibitor YCW1 with antitumor and antimetastasis properties enhances cisplatin activity against non-small cell lung cancer in preclinical studies. Cancer Lett. 346 (1), 84–93. 10.1016/j.canlet.2013.12.016 24355296

[B61] Huang Y. H. Klingbeil O. He X. Y. Wu X. S. Arun G. Lu B. (2018b). POU2F3 is a master regulator of a tuft cell-like variant of small cell lung cancer. Genes. Dev. 32 (13-14), 915–928. 10.1101/gad.314815.118 29945888 PMC6075037

[B62] Huang Y. T. Cheng A. C. Tang H. C. Huang G. C. Cai L. Lin T. H. (2021). USP7 facilitates SMAD3 autoregulation to repress cancer progression in p53-deficient lung cancer. Cell Death Dis. 12 (10), 880. 10.1038/s41419-021-04176-8 34580281 PMC8476631

[B63] Ireland A. S. Micinski A. M. Kastner D. W. Guo B. Wait S. J. Spainhower K. B. (2020). MYC drives temporal evolution of small cell lung cancer subtypes by reprogramming neuroendocrine fate. Cancer Cell 38 (1), 60–78. 10.1016/j.ccell.2020.05.001 32473656 PMC7393942

[B64] Jang J. E. Eom J. I. Jeung H. K. Cheong J. W. Lee J. Y. Kim J. S. (2017). Targeting AMPK-ULK1-mediated autophagy for combating BET inhibitor resistance in acute myeloid leukemia stem cells. Autophagy 13 (4), 761–762. 10.1080/15548627.2016.1278328 28118076 PMC5388226

[B65] Jiang X. Qin N. Hua T. Wei X. Li Y. Chen C. (2022). Functional characterization and clinical significance of super-enhancers in lung adenocarcinoma. Mol. Carcinog. 61 (8), 776–786. 10.1002/mc.23419 35596703

[B66] Jiang Y. Qian F. Bai X. Liu Y. Wang Q. Ai B. (2019). SEdb: a comprehensive human super-enhancer database. Nucleic Acids Res. 47 (D1), D235-D243–d43. 10.1093/nar/gky1025 30371817 PMC6323980

[B67] Jiang Y. Y. Jiang Y. Li C. Q. Zhang Y. Dakle P. Kaur H. (2020). TP63, SOX2, and KLF5 establish a core regulatory circuitry that controls epigenetic and transcription patterns in esophageal squamous cell carcinoma cell lines. Gastroenterology 159 (4), 1311–1327. 10.1053/j.gastro.2020.06.050 32619460

[B68] Kang Y. Kang J. Kim A. (2021). Histone H3K4me1 strongly activates the DNase I hypersensitive sites in super-enhancers than those in typical enhancers. Biosci. Rep. 41 (7). 10.1042/bsr20210691 PMC8264496 34195788

[B69] Khan A. Zhang X. (2016). dbSUPER: a database of super-enhancers in mouse and human genome. Nucleic Acids Res. 44 (D1), D164–D171. 10.1093/nar/gkv1002 26438538 PMC4702767

[B70] Klemm S. L. Shipony Z. Greenleaf W. J. (2019). Chromatin accessibility and the regulatory epigenome. Nat. Rev. Genet. 20 (4), 207–220. 10.1038/s41576-018-0089-8 30675018

[B71] Kling M. J. Kesherwani V. Mishra N. K. Alexander G. McIntyre E. M. Ray S. (2022). A novel dual epigenetic approach targeting BET proteins and HDACs in Group 3 (MYC-driven) Medulloblastoma. J. Exp. Clin. Cancer Res. 41 (1), 321. 10.1186/s13046-022-02530-y 36357906 PMC9650837

[B72] Kong R. Patel A. S. Sato T. Jiang F. Yoo S. Bao L. (2022). Transcriptional circuitry of NKX2-1 and SOX1 defines an unrecognized lineage subtype of small-cell lung cancer. Am. J. Respir. Crit. Care Med. 206 (12), 1480–1494. 10.1164/rccm.202110-2358OC 35848993 PMC9757094

[B73] Kostyrko K. Román M. Lee A. G. Simpson D. R. Dinh P. T. Leung S. G. (2023). UHRF1 is a mediator of KRAS driven oncogenesis in lung adenocarcinoma. Nat. Commun. 14 (1), 3966. 10.1038/s41467-023-39591-2 37407562 PMC10322837

[B74] Kumari A. Gesumaria L. Liu Y. J. Hughitt V. K. Zhang X. Ceribelli M. (2023). mTOR inhibition overcomes RSK3-mediated resistance to BET inhibitors in small cell lung cancer. JCI Insight 8 (5), e156657. 10.1172/jci.insight.156657 36883564 PMC10077471

[B75] Kwiatkowski N. Zhang T. Rahl P. B. Abraham B. J. Reddy J. Ficarro S. B. (2014). Targeting transcription regulation in cancer with a covalent CDK7 inhibitor. Nature 511 (7511), 616–620. 10.1038/nature13393 25043025 PMC4244910

[B76] Lam L. T. Lin X. Faivre E. J. Yang Z. Huang X. Wilcox D. M. (2017). Vulnerability of small-cell lung cancer to apoptosis induced by the combination of BET bromodomain proteins and BCL2 inhibitors. Mol. Cancer Ther. 16 (8), 1511–1520. 10.1158/1535-7163.Mct-16-0459 28468776

[B77] Larochelle S. Amat R. Glover-Cutter K. Sansó M. Zhang C. Allen J. J. (2012). Cyclin-dependent kinase control of the initiation-to-elongation switch of RNA polymerase II. Nat. Struct. Mol. Biol. 19 (11), 1108–1115. 10.1038/nsmb.2399 23064645 PMC3746743

[B78] Lasko L. M. Jakob C. G. Edalji R. P. Qiu W. Montgomery D. Digiammarino E. L. (2017). Discovery of a selective catalytic p300/CBP inhibitor that targets lineage-specific tumours. Nature 550 (7674), 128–132. 10.1038/nature24028 28953875 PMC6050590

[B79] Lee J. H. Xiong F. Li W. (2020). Enhancer RNAs in cancer: regulation, mechanisms and therapeutic potential. RNA Biol. 17 (11), 1550–1559. 10.1080/15476286.2020.1712895 31916476 PMC7567500

[B80] Lengauer C. Kinzler K. W. Vogelstein B. (1998). Genetic instabilities in human cancers. Nature 396 (6712), 643–649. 10.1038/25292 9872311

[B81] Lewin J. Soria J. C. Stathis A. Delord J. P. Peters S. Awada A. (2018). Phase ib trial with Birabresib, a small-molecule inhibitor of bromodomain and extraterminal proteins, in patients with selected advanced solid tumors. J. Clin. Oncol. 36 (30), 3007–3014. 10.1200/jco.2018.78.2292 29733771

[B82] Li F. Fang Z. Zhang J. Li C. Liu H. Xia J. (2016). Identification of TRA2B-DNAH5 fusion as a novel oncogenic driver in human lung squamous cell carcinoma. Cell Res. 26 (10), 1149–1164. 10.1038/cr.2016.111 27670699 PMC5113306

[B83] Li H. Muhetaer G. Xie Y. Yao K. Ma Q. Guan H. (2022a). Identification of super-enhancer-driven peptidyl arginine deiminases as potential biomarkers and therapeutic targets for osimertinib-resistant non-small cell lung cancer. Front. Pharmacol. 13, 1071365. 10.3389/fphar.2022.1071365 36479196 PMC9719927

[B84] Li J. Wang J. Wang Y. Zhao X. Su T. (2023). E2F1 combined with LINC01004 super-enhancer to promote hepatocellular carcinoma cell proliferation and metastasis. Clin. Epigenetics 15 (1), 17. 10.1186/s13148-023-01428-6 36721155 PMC9887888

[B85] Li J. Zhang Y. Wang L. Li M. Yang J. Chen P. (2022b). FOXA1 prevents nutrients deprivation induced autophagic cell death through inducing loss of imprinting of IGF2 in lung adenocarcinoma. Cell Death Dis. 13 (8), 711. 10.1038/s41419-022-05150-8 35974000 PMC9381574

[B86] Li K. Xu C. Du Y. Junaid M. Kaushik A. C. Wei D. Q. (2019a). Comprehensive epigenetic analyses reveal master regulators driving lung metastasis of breast cancer. J. Cell Mol. Med. 23 (8), 5415–5431. 10.1111/jcmm.14424 31215771 PMC6653217

[B87] Li M. Yang B. Li X. Ren H. Zhang L. Li L. (2021). Identification of prognostic factors related to super enhancer-regulated ceRNA network in metastatic lung adenocarcinoma. Int. J. Gen. Med. 14, 6261–6275. 10.2147/ijgm.S332317 34629892 PMC8493278

[B88] Li X. Lu C. Lu Q. Li C. Zhu J. Zhao T. (2019b). Differentiated super-enhancers in lung cancer cells. Sci. China Life Sci. 62 (9), 1218–1228. 10.1007/s11427-018-9319-4 30635833

[B89] Liu C. Qian L. Vallega K. A. Ma G. Zong D. Chen L. (2022b). The novel BET degrader, QCA570, is highly active against the growth of human NSCLC cells and synergizes with osimertinib in suppressing osimertinib-resistant EGFR-mutant NSCLC cells. Am. J. Cancer Res. 12 (2), 779–792. 35261801 PMC8900006

[B90] Liu Q. Guo L. Lou Z. Xiang X. Shao J. (2022a). Super-enhancers and novel therapeutic targets in colorectal cancer. Cell Death Dis. 13 (3), 228. 10.1038/s41419-022-04673-4 35277481 PMC8917125

[B91] Liu Y. Li Y. Liu S. Adeegbe D. O. Christensen C. L. Quinn M. M. (2018). NK cells mediate synergistic antitumor effects of combined inhibition of HDAC6 and BET in a SCLC preclinical model. Cancer Res. 78 (13), 3709–3717. 10.1158/0008-5472.Can-18-0161 29760044 PMC6450092

[B92] Lovén J. Hoke H. A. Lin C. Y. Lau A. Orlando D. A. Vakoc C. R. (2013). Selective inhibition of tumor oncogenes by disruption of super-enhancers. Cell 153 (2), 320–334. 10.1016/j.cell.2013.03.036 23582323 PMC3760967

[B93] Lu H. Xue Y. Yu G. K. Arias C. Lin J. Fong S. (2015). Compensatory induction of MYC expression by sustained CDK9 inhibition via a BRD4-dependent mechanism. Elife 4, e06535. 10.7554/eLife.06535 26083714 PMC4490784

[B94] Luo H. Shan J. Zhang H. Song G. Li Q. Xu C. X. (2022). Targeting the epigenetic processes to enhance antitumor immunity in small cell lung cancer. Semin. Cancer Biol. 86 (Pt 3), 960–970. 10.1016/j.semcancer.2022.02.018 35189321

[B95] Mansour M. R. Abraham B. J. Anders L. Berezovskaya A. Gutierrez A. Durbin A. D. (2014). Oncogene regulation. An oncogenic super-enhancer formed through somatic mutation of a noncoding intergenic element. Science 346 (6215), 1373–1377. 10.1126/science.1259037 25394790 PMC4720521

[B96] Mao R. Wu Y. Ming Y. Xu Y. Wang S. Chen X. (2019). Enhancer RNAs: a missing regulatory layer in gene transcription. Sci. China Life Sci. 62 (7), 905–912. 10.1007/s11427-017-9370-9 30593613

[B97] Martínez-Jiménez F. Muiños F. Sentís I. Deu-Pons J. Reyes-Salazar I. Arnedo-Pac C. (2020). A compendium of mutational cancer driver genes. Nat. Rev. Cancer 20 (10), 555–572. 10.1038/s41568-020-0290-x 32778778

[B98] Miyakawa K. Miyashita N. Horie M. Terasaki Y. Tanaka H. Urushiyama H. (2022). ASCL1 regulates super-enhancer-associated miRNAs to define molecular subtypes of small cell lung cancer. Cancer Sci. 113 (11), 3932–3946. 10.1111/cas.15481 35789143 PMC9633298

[B99] Miyashita N. Horie M. Suzuki H. I. Yoshihara M. Djureinovic D. Persson J. (2018). An integrative analysis of transcriptome and epigenome features of ASCL1-positive lung adenocarcinomas. J. Thorac. Oncol. 13 (11), 1676–1691. 10.1016/j.jtho.2018.07.096 30121393

[B100] Nagaraja S. Vitanza N. A. Woo P. J. Taylor K. R. Liu F. Zhang L. (2017). Transcriptional dependencies in diffuse intrinsic pontine glioma. Cancer Cell 31 (5), 635–652. 10.1016/j.ccell.2017.03.011 28434841 PMC5462626

[B101] Nguyen T. T. T. Zhang Y. Shang E. Shu C. Torrini C. Zhao J. (2020). HDAC inhibitors elicit metabolic reprogramming by targeting super-enhancers in glioblastoma models. J. Clin. Invest. 130 (7), 3699–3716. 10.1172/jci129049 32315286 PMC7324177

[B102] Ogryzko V. V. Schiltz R. L. Russanova V. Howard B. H. Nakatani Y. (1996). The transcriptional coactivators p300 and CBP are histone acetyltransferases. Cell 87 (5), 953–959. 10.1016/s0092-8674(00)82001-2 8945521

[B103] Parker S. C. Stitzel M. L. Taylor D. L. Orozco J. M. Erdos M. R. Akiyama J. A. (2013). Chromatin stretch enhancer states drive cell-specific gene regulation and harbor human disease risk variants. Proc. Natl. Acad. Sci. U. S. A. 110 (44), 17921–17926. 10.1073/pnas.1317023110 24127591 PMC3816444

[B104] Pastore A. Gaiti F. Lu S. X. Brand R. M. Kulm S. Chaligne R. (2019). Corrupted coordination of epigenetic modifications leads to diverging chromatin states and transcriptional heterogeneity in CLL. Nat. Commun. 10 (1), 1874. 10.1038/s41467-019-09645-5 31015400 PMC6478836

[B105] Peng L. Jiang B. Yuan X. Qiu Y. Peng J. Huang Y. (2019). Super-enhancer-associated long noncoding RNA HCCL5 is activated by ZEB1 and promotes the malignancy of hepatocellular carcinoma. Cancer Res. 79 (3), 572–584. 10.1158/0008-5472.Can-18-0367 30482773

[B106] Persson M. Andrén Y. Mark J. Horlings H. M. Persson F. Stenman G. (2009). Recurrent fusion of MYB and NFIB transcription factor genes in carcinomas of the breast and head and neck. Proc. Natl. Acad. Sci. U. S. A. 106 (44), 18740–18744. 10.1073/pnas.0909114106 19841262 PMC2773970

[B107] Pongor L. S. Tlemsani C. Elloumi F. Arakawa Y. Jo U. Gross J. M. (2022). Integrative epigenomic analyses of small cell lung cancer cells demonstrates the clinical translational relevance of gene body methylation. iScience 25 (11), 105338. 10.1016/j.isci.2022.105338 36325065 PMC9619308

[B108] Pozo K. Kollipara R. K. Kelenis D. P. Rodarte K. E. Ullrich M. S. Zhang X. (2021). ASCL1, NKX2-1, and PROX1 co-regulate subtype-specific genes in small-cell lung cancer. iScience 24 (9), 102953. 10.1016/j.isci.2021.102953 34466783 PMC8384902

[B109] Qian F. C. Li X. C. Guo J. C. Zhao J. M. Li Y. Y. Tang Z. D. (2019). SEanalysis: a web tool for super-enhancer associated regulatory analysis. Nucleic Acids Res. 47 (W1), W248-W255–w55. 10.1093/nar/gkz302 31028388 PMC6602466

[B110] Qu S. Fetsch P. Thomas A. Pommier Y. Schrump D. S. Miettinen M. M. (2022). Molecular subtypes of primary SCLC tumors and their associations with neuroendocrine and therapeutic markers. J. Thorac. Oncol. 17 (1), 141–153. 10.1016/j.jtho.2021.08.763 34534680 PMC8692365

[B111] Reck M. Horn L. Novello S. Barlesi F. Albert I. Juhász E. (2019). Phase II study of roniciclib in combination with cisplatin/etoposide or carboplatin/etoposide as first-line therapy in patients with extensive-disease small cell lung cancer. J. Thorac. Oncol. 14 (4), 701–711. 10.1016/j.jtho.2019.01.010 30677506

[B112] Rengachari S. Schilbach S. Aibara S. Dienemann C. Cramer P. (2021). Structure of the human Mediator-RNA polymerase II pre-initiation complex. Nature 594 (7861), 129–133. 10.1038/s41586-021-03555-7 33902108

[B113] Riveiro M. E. Astorgues-Xerri L. Vazquez R. Frapolli R. Kwee I. Rinaldi A. (2016). OTX015 (MK-8628), a novel BET inhibitor, exhibits antitumor activity in non-small cell and small cell lung cancer models harboring different oncogenic mutations. Oncotarget 7 (51), 84675–84687. 10.18632/oncotarget.13181 27835869 PMC5354535

[B114] Roden A. C. Greipp P. T. Knutson D. L. Kloft-Nelson S. M. Jenkins S. M. Marks R. S. (2015). Histopathologic and cytogenetic features of pulmonary adenoid cystic carcinoma. J. Thorac. Oncol. 10 (11), 1570–1575. 10.1097/jto.0000000000000656 26309189

[B115] Ropri A. S. DeVaux R. S. Eng J. Chittur S. V. Herschkowitz J. I. (2021). Cis-acting super-enhancer lncRNAs as biomarkers to early-stage breast cancer. Breast Cancer Res. 23 (1), 101. 10.1186/s13058-021-01479-8 34717732 PMC8557595

[B116] Rudin C. M. Poirier J. T. Byers L. A. Dive C. Dowlati A. George J. (2019). Molecular subtypes of small cell lung cancer: a synthesis of human and mouse model data. Nat. Rev. Cancer 19 (5), 289–297. 10.1038/s41568-019-0133-9 30926931 PMC6538259

[B117] Sabari B. R. Dall'Agnese A. Boija A. Klein I. A. Coffey E. L. Shrinivas K. (2018). Coactivator condensation at super-enhancers links phase separation and gene control. Science 361 (6400), eaar3958. 10.1126/science.aar3958 29930091 PMC6092193

[B118] Saint-André V. Federation A. J. Lin C. Y. Abraham B. J. Reddy J. Lee T. I. (2016). Models of human core transcriptional regulatory circuitries. Genome Res. 26 (3), 385–396. 10.1101/gr.197590.115 26843070 PMC4772020

[B119] Sanchez G. J. Richmond P. A. Bunker E. N. Karman S. S. Azofeifa J. Garnett A. T. (2018). Genome-wide dose-dependent inhibition of histone deacetylases studies reveal their roles in enhancer remodeling and suppression of oncogenic super-enhancers. Nucleic Acids Res. 46 (4), 1756–1776. 10.1093/nar/gkx1225 29240919 PMC5829637

[B120] Sato T. Yoo S. Kong R. Sinha A. Chandramani-Shivalingappa P. Patel A. (2019). Epigenomic profiling discovers trans-lineage SOX2 partnerships driving tumor heterogeneity in lung squamous cell carcinoma. Cancer Res. 79 (24), 6084–6100. 10.1158/0008-5472.Can-19-2132 31551362 PMC6911633

[B121] Schaukowitch K. Joo J. Y. Liu X. Watts J. K. Martinez C. Kim T. K. (2014). Enhancer RNA facilitates NELF release from immediate early genes. Mol. Cell 56 (1), 29–42. 10.1016/j.molcel.2014.08.023 25263592 PMC4186258

[B122] Shen Q. Sun Y. Xu S. (2020). LINC01503/miR-342-3p facilitates malignancy in non-small-cell lung cancer cells via regulating LASP1. Respir. Res. 21 (1), 235. 10.1186/s12931-020-01464-3 32938459 PMC7493870

[B123] Shi K. Yin X. Cai M. C. Yan Y. Jia C. Ma P. (2019). PAX8 regulon in human ovarian cancer links lineage dependency with epigenetic vulnerability to HDAC inhibitors. Elife 8, e44306. 10.7554/eLife.44306 31050342 PMC6533083

[B124] Shi T. Hu Z. Tian L. Yang Y. (2023). Advances in lung adenocarcinoma: a novel perspective on prognoses and immune responses of CENPO as an oncogenic superenhancer. Transl. Oncol. 34, 101691. 10.1016/j.tranon.2023.101691 37207381 PMC10209335

[B125] Shi Y. Wang M. Liu D. Ullah S. Ma X. Yang H. (2022). Super-enhancers in esophageal carcinoma: transcriptional addictions and therapeutic strategies. Front. Oncol. 12, 1036648. 10.3389/fonc.2022.1036648 36387198 PMC9647064

[B126] Siegel R. L. Miller K. D. Fuchs H. E. Jemal A. (2022). Cancer statistics, 2022. CA Cancer J. Clin. 72 (1), 7–33. 10.3322/caac.21708 35020204

[B127] Siegel R. L. Miller K. D. Jemal A. (2020). Cancer statistics, 2020. CA Cancer J. Clin. 70 (1), 7–30. 10.3322/caac.21590 31912902

[B128] Soibam B. (2017). Super-lncRNAs: identification of lncRNAs that target super-enhancers via RNA:DNA:DNA triplex formation. Rna 23 (11), 1729–1742. 10.1261/rna.061317.117 28839111 PMC5648039

[B129] Song X. Zhang T. Ding H. Feng Y. Yang W. Yin X. (2022). Non-genetic stratification reveals epigenetic heterogeneity and identifies vulnerabilities of glycolysis addiction in lung adenocarcinoma subtype. Oncogenesis 11 (1), 61. 10.1038/s41389-022-00436-0 36216804 PMC9550819

[B130] Spitz F. Furlong E. E. (2012). Transcription factors: from enhancer binding to developmental control. Nat. Rev. Genet. 13 (9), 613–626. 10.1038/nrg3207 22868264

[B131] Stephenson J. J. Nemunaitis J. Joy A. A. Martin J. C. Jou Y. M. Zhang D. (2014). Randomized phase 2 study of the cyclin-dependent kinase inhibitor dinaciclib (MK-7965) versus erlotinib in patients with non-small cell lung cancer. Lung Cancer 83 (2), 219–223. 10.1016/j.lungcan.2013.11.020 24388167

[B132] Su T. Zhang N. Wang T. Zeng J. Li W. Han L. (2023). Super enhancer-regulated LncRNA LINC01089 induces alternative splicing of DIAPH3 to drive hepatocellular carcinoma metastasis. Cancer Res. 83 (24), 4080–4094. 10.1158/0008-5472.Can-23-0544 37756562

[B133] Sun D. Nikonova A. S. Zhang P. Deneka A. Y. Fitzgerald M. E. Michael R. E. (2021). Evaluation of the small-molecule BRD4 degrader CFT-2718 in small-cell lung cancer and pancreatic cancer models. Mol. Cancer Ther. 20 (8), 1367–1377. 10.1158/1535-7163.Mct-20-0831 34045230 PMC8338762

[B134] Suski J. M. Braun M. Strmiska V. Sicinski P. (2021). Targeting cell-cycle machinery in cancer. Cancer Cell 39 (6), 759–778. 10.1016/j.ccell.2021.03.010 33891890 PMC8206013

[B135] Takashima Y. Kikuchi E. Kikuchi J. Suzuki M. Kikuchi H. Maeda M. (2020). Bromodomain and extraterminal domain inhibition synergizes with WEE1-inhibitor AZD1775 effect by impairing nonhomologous end joining and enhancing DNA damage in nonsmall cell lung cancer. Int. J. Cancer 146 (4), 1114–1124. 10.1002/ijc.32515 31199520

[B136] Tien F. M. Lu H. H. Lin S. Y. Tsai H. C. (2023). Epigenetic remodeling of the immune landscape in cancer: therapeutic hurdles and opportunities. J. Biomed. Sci. 30 (1), 3. 10.1186/s12929-022-00893-0 36627707 PMC9832644

[B137] Trabucco S. E. Gerstein R. M. Evens A. M. Bradner J. E. Shultz L. D. Greiner D. L. (2015). Inhibition of bromodomain proteins for the treatment of human diffuse large B-cell lymphoma. Clin. Cancer Res. 21 (1), 113–122. 10.1158/1078-0432.Ccr-13-3346 25009295 PMC4286476

[B138] Travis W. D. Brambilla E. Nicholson A. G. Yatabe Y. Austin J. H. M. Beasley M. B. (2015). The 2015 world health organization classification of lung tumors: impact of genetic, clinical and radiologic advances since the 2004 classification. J. Thorac. Oncol. 10 (9), 1243–1260. 10.1097/jto.0000000000000630 26291008

[B139] Traynor A. M. Dubey S. Eickhoff J. C. Kolesar J. M. Schell K. Huie M. S. (2009). Vorinostat (NSC# 701852) in patients with relapsed non-small cell lung cancer: a Wisconsin Oncology Network phase II study. J. Thorac. Oncol. 4 (4), 522–526. 10.1097/jto.0b013e3181952478 19347984 PMC3050710

[B140] Trojanowski J. Frank L. Rademacher A. Mücke N. Grigaitis P. Rippe K. (2022). Transcription activation is enhanced by multivalent interactions independent of phase separation. Mol. Cell 82 (10), 1878–1893.e10. 10.1016/j.molcel.2022.04.017 35537448

[B141] Tu Y. H. Juan H. F. Huang H. C. (2021). Identification of cell states using super-enhancer RNA. BMC Genomics 22 (Suppl. 3), 787. 10.1186/s12864-021-08092-1 34727867 PMC8564956

[B142] Vahedi G. Kanno Y. Furumoto Y. Jiang K. Parker S. C. Erdos M. R. (2015). Super-enhancers delineate disease-associated regulatory nodes in T cells. Nature 520 (7548), 558–562. 10.1038/nature14154 25686607 PMC4409450

[B143] Wang H. Hong B. Li X. Deng K. Li H. Yan Lui V. W. (2017). JQ1 synergizes with the Bcl-2 inhibitor ABT-263 against MYCN-amplified small cell lung cancer. Oncotarget 8 (49), 86312–86324. 10.18632/oncotarget.21146 29156797 PMC5689687

[B144] Wang J. Liu J. Li S. Li X. Yang J. Dang X. (2023b). Genetic regulatory and biological implications of the 10q24.32 schizophrenia risk locus. Brain 146 (4), 1403–1419. 10.1093/brain/awac352 36152315 PMC10115178

[B145] Wang M. Chen Z. Zhang Y. (2022). CBP/p300 and HDAC activities regulate H3K27 acetylation dynamics and zygotic genome activation in mouse preimplantation embryos. Embo J. 41 (22), e112012. 10.15252/embj.2022112012 36215692 PMC9670200

[B146] Wang M. Herbst R. S. Boshoff C. (2021). Toward personalized treatment approaches for non-small-cell lung cancer. Nat. Med. 27 (8), 1345–1356. 10.1038/s41591-021-01450-2 34385702

[B147] Wang X. Yu C. Wang C. Ma Y. Wang T. Li Y. (2019). Novel cyclin-dependent kinase 9 (CDK9) inhibitor with suppression of cancer stemness activity against non-small-cell lung cancer. Eur. J. Med. Chem. 181, 111535. 10.1016/j.ejmech.2019.07.038 31376566

[B148] Wang Y. Song C. Zhao J. Zhang Y. Zhao X. Feng C. (2023a). SEdb 2.0: a comprehensive super-enhancer database of human and mouse. Nucleic Acids Res. 51 (D1), D280–D290. 10.1093/nar/gkac968 36318264 PMC9825585

[B149] Wang Y. Zhang T. Kwiatkowski N. Abraham B. J. Lee T. I. Xie S. (2015). CDK7-dependent transcriptional addiction in triple-negative breast cancer. Cell 163 (1), 174–186. 10.1016/j.cell.2015.08.063 26406377 PMC4583659

[B150] Watanabe H. Ma Q. Peng S. Adelmant G. Swain D. Song W. (2014). SOX2 and p63 colocalize at genetic loci in squamous cell carcinomas. J. Clin. Invest. 124 (4), 1636–1645. 10.1172/jci71545 24590290 PMC3973117

[B151] Wei J. Yu H. Liu T. Wang Z. Lang C. Pan Y. (2023). FOXA1-induced LINC00621 promotes lung adenocarcinoma progression via activating the TGF-β signaling pathway. Thorac. Cancer 14 (21), 2026–2037. 10.1111/1759-7714.14986 37277890 PMC10363846

[B152] Wei Y. Zhang S. Shang S. Zhang B. Li S. Wang X. (2016). SEA: a super-enhancer archive. Nucleic Acids Res. 44 (D1), D172–D179. 10.1093/nar/gkv1243 26578594 PMC4702879

[B153] Whyte W. A. Orlando D. A. Hnisz D. Abraham B. J. Lin C. Y. Kagey M. H. (2013). Master transcription factors and mediator establish super-enhancers at key cell identity genes. Cell 153 (2), 307–319. 10.1016/j.cell.2013.03.035 23582322 PMC3653129

[B154] Wiese M. Hamdan F. H. Kubiak K. Diederichs C. Gielen G. H. Nussbaumer G. (2020). Combined treatment with CBP and BET inhibitors reverses inadvertent activation of detrimental super enhancer programs in DIPG cells. Cell Death Dis. 11 (8), 673. 10.1038/s41419-020-02800-7 32826850 PMC7442654

[B155] William W. N. Jr. Glisson B. S. (2011). Novel strategies for the treatment of small-cell lung carcinoma. Nat. Rev. Clin. Oncol. 8 (10), 611–619. 10.1038/nrclinonc.2011.90 21691321

[B156] Wu L. Yao H. Chen H. Wang A. Guo K. Gou W. (2022). Landscape of somatic alterations in large-scale solid tumors from an Asian population. Nat. Commun. 13 (1), 4264. 10.1038/s41467-022-31780-9 35871175 PMC9308789

[B157] Wu P. F. Gao W. W. Sun C. L. Ma T. Hao J. Q. (2020). Suberoylanilide hydroxamic acid overcomes erlotinib-acquired resistance via phosphatase and tensin homolog deleted on chromosome 10-mediated apoptosis in non-small cell lung cancer. Chin. Med. J. Engl. 133 (11), 1304–1311. 10.1097/cm9.0000000000000823 32452893 PMC7289310

[B158] Wu Y. Chen S. Shao Y. Su Y. Li Q. Wu J. (2023). KLF5 promotes tumor progression and parp inhibitor resistance in ovarian cancer. Adv. Sci. (Weinh) 10 (31), e2304638. 10.1002/advs.202304638 37702443 PMC10625120

[B159] Xie J. J. Jiang Y. Y. Jiang Y. Li C. Q. Lim M. C. An O. (2018). Super-enhancer-driven long non-coding RNA LINC01503, regulated by TP63, is over-expressed and oncogenic in squamous cell carcinoma. Gastroenterology 154 (8), 2137–2151. 10.1053/j.gastro.2018.02.018 29454790

[B160] Xie K. Feng J. Fan D. Wang S. Luo J. Ren Z. (2022). BARX2/FOXA1/HK2 axis promotes lung adenocarcinoma progression and energy metabolism reprogramming. Transl. Lung Cancer Res. 11 (7), 1405–1419. 10.21037/tlcr-22-465 35958341 PMC9359959

[B161] Xing R. Zhou Y. Yu J. Yu Y. Nie Y. Luo W. (2019). Whole-genome sequencing reveals novel tandem-duplication hotspots and a prognostic mutational signature in gastric cancer. Nat. Commun. 10 (1), 2037. 10.1038/s41467-019-09644-6 31048690 PMC6497673

[B162] Xu Y. Wu Y. Zhang S. Ma P. Jin X. Wang Z. (2019). A tumor-specific super-enhancer drives immune evasion by guiding synchronous expression of PD-L1 and PD-L2. Cell Rep. 29 (11), 3435–3447. 10.1016/j.celrep.2019.10.093 31825827

[B163] Yamagata K. Nakayamada S. Zhang T. Nguyen A. P. Ohkubo N. Iwata S. (2022). IL-6 production through repression of UBASH3A gene via epigenetic dysregulation of super-enhancer in CD4(+) T cells in rheumatoid arthritis. Inflamm. Regen. 42 (1), 46. 10.1186/s41232-022-00231-9 36324153 PMC9632101

[B164] Yan L. Chen H. Tang L. Jiang P. Yan F. (2021). Super-enhancer-associated long noncoding RNA AC005592.2 promotes tumor progression by regulating OLFM4 in colorectal cancer. BMC Cancer 21 (1), 187. 10.1186/s12885-021-07900-x 33622275 PMC7903608

[B165] Yang J. Xu J. Wang W. Zhang B. Yu X. Shi S. (2023a). Epigenetic regulation in the tumor microenvironment: molecular mechanisms and therapeutic targets. Signal Transduct. Target Ther. 8 (1), 210. 10.1038/s41392-023-01480-x 37217462 PMC10203321

[B166] Yang X. Han H. De Carvalho D. D. Lay F. D. Jones P. A. Liang G. (2014). Gene body methylation can alter gene expression and is a therapeutic target in cancer. Cancer Cell 26 (4), 577–590. 10.1016/j.ccr.2014.07.028 25263941 PMC4224113

[B167] Yang Z. Yik J. H. Chen R. He N. Jang M. K. Ozato K. (2005). Recruitment of P-TEFb for stimulation of transcriptional elongation by the bromodomain protein Brd4. Mol. Cell 19 (4), 535–545. 10.1016/j.molcel.2005.06.029 16109377

[B168] Yang Z. Zheng Y. Wu H. Xie H. Zhao J. Chen Z. (2023b). Integrative analysis of a novel super-enhancer-associated lncRNA prognostic signature and identifying LINC00945 in aggravating glioma progression. Hum. Genomics 17 (1), 33. 10.1186/s40246-023-00480-w 37004060 PMC10064652

[B169] Yoo K. H. Hennighausen L. Shin H. Y. (2019). Dissecting tissue-specific super-enhancers by integrating genome-wide analyses and CRISPR/Cas9 genome editing. J. Mammary Gland. Biol. Neoplasia 24 (1), 47–59. 10.1007/s10911-018-9417-z 30291498

[B170] You L. Guo X. Huang Y. (2018). Correlation of cancer stem-cell markers OCT4, SOX2, and NANOG with clinicopathological features and prognosis in operative patients with rectal cancer. Yonsei Med. J. 59 (1), 35–42. 10.3349/ymj.2018.59.1.35 29214774 PMC5725361

[B171] Yuan C. Chen H. Tu S. Huang H. Y. Pan Y. Gui X. (2021). A systematic dissection of the epigenomic heterogeneity of lung adenocarcinoma reveals two different subclasses with distinct prognosis and core regulatory networks. Genome Biol. 22 (1), 156. 10.1186/s13059-021-02376-1 34001209 PMC8127276

[B172] Yuan X. Q. Zhou N. Wang J. P. Yang X. Z. Wang S. Zhang C. Y. (2023). Anchoring super-enhancer-driven oncogenic lncRNAs for anti-tumor therapy in hepatocellular carcinoma. Mol. Ther. 31 (6), 1756–1774. 10.1016/j.ymthe.2022.11.013 36461633 PMC10277835

[B173] Zhang J. Zhang X. Xie F. Zhang Z. van Dam H. Zhang L. (2014). The regulation of TGF-β/SMAD signaling by protein deubiquitination. Protein Cell 5 (7), 503–517. 10.1007/s13238-014-0058-8 24756567 PMC4085288

[B174] Zhang T. Song X. Zhang Z. Mao Q. Xia W. Xu L. (2020a). Aberrant super-enhancer landscape reveals core transcriptional regulatory circuitry in lung adenocarcinoma. Oncogenesis 9 (10), 92. 10.1038/s41389-020-00277-9 33070167 PMC7568720

[B175] Zhang T. Xia W. Song X. Mao Q. Huang X. Chen B. (2022). Super-enhancer hijacking LINC01977 promotes malignancy of early-stage lung adenocarcinoma addicted to the canonical TGF-β/SMAD3 pathway. J. Hematol. Oncol. 15 (1), 114. 10.1186/s13045-022-01331-2 35982471 PMC9389757

[B176] Zhang X. Choi P. S. Francis J. M. Imielinski M. Watanabe H. Cherniack A. D. (2016). Identification of focally amplified lineage-specific super-enhancers in human epithelial cancers. Nat. Genet. 48 (2), 176–182. 10.1038/ng.3470 26656844 PMC4857881

[B177] Zhang Y. Huang Y. X. Wang D. L. Yang B. Yan H. Y. Lin L. H. (2020b). LncRNA DSCAM-AS1 interacts with YBX1 to promote cancer progression by forming a positive feedback loop that activates FOXA1 transcription network. Theranostics 10 (23), 10823–10837. 10.7150/thno.47830 32929382 PMC7482804

[B178] Zheng J. Huang B. Xiao L. Wu M. (2024). Effects of BRD4 inhibitor JQ1 on the expression profile of super-enhancer related lncRNAs and mRNAs in cervical cancer HeLa cells. PeerJ 12, e17035. 10.7717/peerj.17035 38410799 PMC10896078

[B179] Zhou J. Wang D. Tang D. Huang W. (2020). Abnormal activations of super-enhancers enhance the carcinogenicity in lung adenocarcinoma. Cancer Manag. Res. 12, 8509–8518. 10.2147/cmar.S258497 32982443 PMC7501973

[B180] Zhou Q. Li T. Price D. H. (2012). RNA polymerase II elongation control. Annu. Rev. Biochem. 81, 119–143. 10.1146/annurev-biochem-052610-095910 22404626 PMC4273853

[B181] Zhou R. W. Parsons R. E. (2023). Etiology of super-enhancer reprogramming and activation in cancer. Epigenetics Chromatin 16 (1), 29. 10.1186/s13072-023-00502-w 37415185 PMC10324276

[B182] Zhou X. Sun T. Meng Y. Luo J. Chen J. Xia B. (2021). BET inhibitors combined with chemotherapy synergistically inhibit the growth of NSCLC cells. Oncol. Rep. 45 (5), 70. 10.3892/or.2021.8021 33760217

